# LncRNA evf‐2 Exacerbates Podocyte Injury in Diabetic Nephropathy by Inducing Cell Cycle Re‐entry and Inflammation Through Distinct Mechanisms Triggered by hnRNPU

**DOI:** 10.1002/advs.202406532

**Published:** 2024-10-29

**Authors:** Chaojie Zhang, Hui Zhao, Yufan Yan, Yanfei Li, Min Lei, Yong Liu, Longhua Yang, Huijian Zhao, Sijie Zhou, Shaokang Pan, Zhangsuo Liu, Jia Guo

**Affiliations:** ^1^ Nephrology Research Center the First Affiliated Hospital of Zhengzhou University Zhengzhou 450052 P. R. China; ^2^ Henan Province Research Center for Kidney Disease Zhengzhou 450052 P. R. China; ^3^ Key Laboratory of Precision Diagnosis and Treatment for Chronic Kidney Disease in Henan Province Zhengzhou 450052 P. R. China; ^4^ School of Pharmaceutical Sciences & Key Laboratory of Advanced Drug Preparation Technologies Zhengzhou University Henan 450001 China; ^5^ Tianjian Laboratory of Advanced Biomedical Sciences, Academy of Medical Sciences Zhengzhou University Zhengzhou Henan 450001 China; ^6^ Innovation Center of Basic Research for Metabolic‐Associated Fatty Liver Disease Ministry of Education of China China

**Keywords:** Albuminuria, Cell cycle re‐entry, Diabetic nephropathy, Evf‐2, Inflammation, Podocyte

## Abstract

Albuminuria is a hallmark of diabetic nephropathy (DN). Podocyte injury significantly contributes to proteinuria in DN. Our study found that lncRNA EVF‐2 is upregulated in podocytes of DN patients, correlating with cell cycle re‐entry and inflammation. Specific knockout or knockdown of lncRNA evf‐2 in diabetic mice or cultured podocytes alleviated podocyte injury associated with these processes. RNA sequencing of evf‐2‐overexpressing podocytes unveiled a predominant enrichment of upregulated mRNAs in cell cycle and inflammation pathways, with alternative splicing in cell cycle‐related mRNAs Ccnb1 and Tacc3. Chromatin isolation by RNA purification‐mass spectrometry (ChIRP‐MS) analysis highlighted the involvement of ribonucleoprotein complex and mRNA processing‐related proteins, with hnRNPU as the main binding partner of evf‐2 in spliceosomes. Knockdown of hnRNPU partially restored the upregulation of mRNAs induced by evf‐2 overexpression, altering splice variants of Ccnb1 and Tacc3. This study is the first to reveal the splice variants of cell cycle‐related genes in DN and elucidate the interaction between lncRNA evf‐2 and hnRNPU. This interaction culminates in the upregulation of cell cycle‐related genes and inflammatory factors through diverse pathways, potentially involving transcriptional activation, RNA stability modulation, alternative splicing or translational regulation. This highlights potential novel pathways for DN treatment.

## Graphical abstract

Podocyte injury is central to the onset and progression of diabetic nephropathy. This study suggests that lncRNA evf‐2 intricately regulates the transcription, RNA stability, or alternative splicing of inflammatory factors or cell cycle‐related genes by binding to hnRNPU. LncRNA evf‐2 represents a crucial target for addressing podocyte injury, providing a potential way for gene therapy in the future.

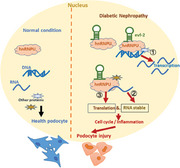

## 1 Introduction

Diabetic nephropathy (DN) stands as one of the most prevalent microvascular complications of diabetes, emerging as the foremost cause of end‐stage renal disease, with its incidence steadily increasing each year.^[^ ^1^ ^]^ Notably, albuminuria represents the predominant clinical manifestation of this complication,^[^ ^2^ ^]^ yet current therapeutic methods remain ineffective.^[^ ^3^ ^]^ Podocytes, specialized visceral epithelial cells terminally differentiated, inhabit the glomerular basement membrane exterior to the glomerular capillaries. Integral components of the glomerular filtration barrier, the intricate foot processes of podocytes, and the slit diaphragm are essential, with their compromise serving as a pivotal factor leading to the development of albuminuria,^[^ ^4^, ^5^ ^]^ further exacerbating damage to other intrinsic renal cell populations.^[^ ^6^ ^]^ Perturbations affecting the podocyte cytoskeleton, such as cell cycle re‐entry, contribute to podocyte injury.^[^ ^7^ ^]^ Moreover, inflammation is recognized as a pivotal pathogenic mechanism underlying podocyte injury in DN.^[^ ^8^ ^]^ Podocytes, with their immune cell‐like functionalities, serve as a crucial link between sterile inflammation and DN. Our previous research has also identified podocyte inflammation in DN patients, elucidating the regulatory roles of RNA binding proteins in modulating chemokine and cytokine expression.^[^ ^9^ ^]^ Notably, targeting inflammation has been deemed a promising therapeutic strategy of diabetic kidney disease in 2030.^[^ ^10^ ^]^ Despite the widespread use of novel therapeutic agents such as sodium‐glucose transport protein 2, glucagon‐like peptide‐2 antagonists, and nonsteroidal mineralocorticoid antagonists,^[^ ^11^ ^]^ there remains an ongoing imperative to develop innovative therapeutic approaches targeting podocyte function, with particular emphasis on addressing cell cycle dysregulation and inflammation of DN.

A growing body of evidence indicates that long non‐coding RNAs (lncRNAs) can interact with DNAs, RNAs, and proteins,^[^ ^12^, ^13^ ^]^ thereby influencing the pathogenesis and progression of DN by regulating gene expression across various levels, including epigenetic, transcriptional, and post‐transcriptional mechanisms.^[^ ^14^ ^]^ However, the functional roles of most lncRNAs remain poorly understood.^[^ ^15^ ^]^ Our research team employed the high‐throughput Arraystar Human LncRNA Chip V3.0 (Shanghai Kangcheng Biotechnology Co., Ltd.) to assess kidney tissue specimens, serum, and urine collected from patients with DN.^[^ ^16^ ^]^ Comparative analyses were conducted among specimens from the DN group, diabetes mellitus (DM) group, and control group. The differential expression profile of lncRNAs in DN patients was obtained. Notably, we observed a significant upregulation in the expression of the lncRNA evf‐2 (Gene ID: 285 987, corresponding to mouse Gene ID: 320 038) in both urine samples and kidney tissues of DN patients, as opposed to DM patients.

As per previous reports, evf‐2 stands out as the first identified lncRNA capable of regulating the expression of crucial proteins during vertebrate organogenesis, thereby assuming a critical role in maintaining normal organ development and function.^[^ ^17^ ^]^ Furthermore, evf‐2 is transcribed from the ultraconserved region of Dlx‐5/6, members of the Dlx/dll homeodomain‐containing protein family, and functions as a transcriptional coactivator for Dlx‐2.^[^ ^18^ ^]^ Consequently, evf‐2 emerges as a key determinant in preserving organ physiology, with dysregulation in its expression levels implicated in disease pathogenesis. Despite its significance, research on evf‐2 remains relatively limited, primarily focusing on its involvement in neurological disorders and tumorigenesis.^[^ ^19^ ^]^ Notably, the potential contribution of evf‐2 to the pathogenesis and progression of kidney diseases, particularly DN, remains largely unexplored.

Heterogeneous nuclear ribonucleoprotein U (hnRNPU), distinguished by its strong affinity for RNA, DNA, and diverse proteins, assumes multifaceted roles in cellular functions including RNA splicing, maintenance of RNA stability, regulation of gene transcription, and organization of chromatin.^[^ ^20^, ^21^ ^]^ However, the precise mechanisms underlying hnRNPU's involvement in regulating the podocyte cell cycle and inflammation remain inadequately characterized.

Our study uncovered a novel interaction between the lncRNA evf‐2 and hnRNPU, which exerts regulatory effects on podocytes' cell cycle re‐entry and inflammatory responses through diverse mechanisms. This regulation involves transcriptional control of inflammatory factors and transcriptional and alternative splicing control of cell cycle‐related proteins, with potential implications on their translation processes, particularly under conditions mimicking DN or exposure to lipopolysaccharides (LPS). Our findings deepen the understanding of the dual regulatory role played by lncRNA evf‐2 and hnRNPU complexes at both DNA and RNA levels in the context of DN albuminuria and other podocytopathy kidney diseases. Importantly, these insights lay the groundwork for developing innovative therapeutic approaches targeting podocyte injury.

## 2 Results:

### 2.1 The Positive Correlation between Elevated lncRNA Evf‐2 Expression and Podocyte Injury in Diabetic Nephropathy

**Figure 1:**
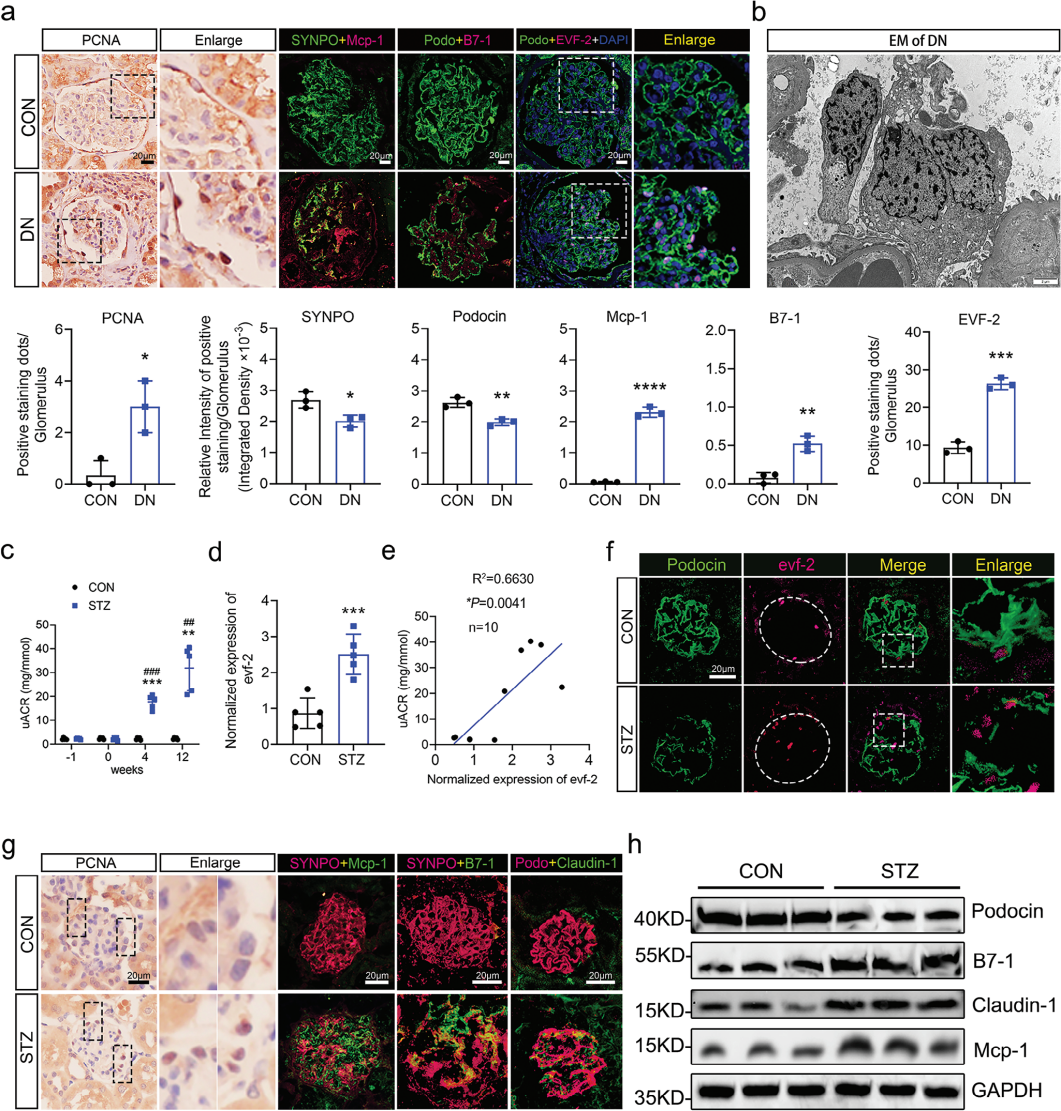
Positive correlation between increased evf‐2 expression and podocyte injury in DN patients and STZ‐induced mice models. a) Paraffin‐embedded renal biopsies were used for immunohistochemistry staining of proliferating cell nuclear antigen (PCNA). Immunofluorescence images of Podocin (green), SYNPO (green), and B7‐1 (magenta), Mcp‐1 (magenta), EVF‐2 (magenta dots) in DN kidney tissue specimens. Bar = 20 µm. An enlarged section (dashed rectangle) is presented. Staining intensity was quantified and compared across samples (n = 3). *p < 0.05, ** < 0.01, *** < 0.001, **** < 0.0001 (unpaired t‐test). b) Representative transmission electron microscopy (EM) micrograph illustrating binucleated podocytes in biopsies from DN patients. Bar = 2 µm. c) The urinary albumin/creatinine ratio (uACR) of mice in the STZ group is significantly higher than that of mice in the CON group. **p < 0.01, *** < 0.001 (STZ versus CON), ##*p* < 0.01, ### < 0.001 (different time points in the STZ group), (two‐way ANOVA plus Dunnett's multiple comparisons test). n = 5 mice/group. d) qRT‐PCR analysis of evf‐2 expression in isolated primary podocytes reveals significant increases in STZ‐induced mice. ***p < 0.001 (unpaired t‐test). n = 5 mice/group. e) A positive correlation between uACR and evf‐2 expression. f) Fluorescence in situ hybridization (FISH)‐based detection of evf‐2 expression levels and localization in paraffin‐embedded kidneys of STZ‐induced mice. Bar = 20 µm. g) Immunohistochemistry staining for PCNA of STZ‐induced mice glomeruli. Immunofluorescence images of Podocin (magenta), SYNPO (magenta), Claudin‐1 (green), B7‐1 (green), and Mcp‐1 (green) in kidney tissue specimens from STZ‐induced mice. Bar = 20 µm. h) Western blots of Podocin, B7‐1, Claudin‐1, and Mcp‐1 in isolated glomeruli from mouse kidney. GAPDH serves as the loading control.

To investigate the correlation between lncRNA evf‐2 expression and podocyte injury in DN, we analyzed evf‐2 expression levels alongside podocyte marker proteins, podocyte injury factors, and inflammatory cytokines (**Figure** **1**a). In DN patients, we observed a significant upregulation of evf‐2 expression. Corresponding to this upregulation, there was a notable increase in the expression of inflammatory factors, such as Mcp‐1 and B7‐1, along with a decrease in the expression of podocyte marker proteins, including podocin and synaptopodin (SYNPO). Additionally, immunohistochemical analysis revealed an enhanced expression of proliferating cell nuclear antigen (PCNA) in the glomeruli of DN patients, indicating the re‐entry of some podocytes into the cell cycle.^[^ ^22^ ^]^ Furthermore, a quantitative analysis provided additional insights into the observed changes. The results are presented below Figure 1a.

In addition to the quantitative analysis, podocyte splitting was re‐evaluated using transmission electron microscopy (EM). The results revealed binucleated podocytes in biopsies from DN patients (Figure 1b). This microscopic observation provides additional evidence of structural changes within podocytes, indicating a potential link between elevated evf‐2 expression and alternations in podocyte morphology in DN patients.

To further elucidate the role of evf‐2 in the development of DN, a streptozotocin (STZ) injection combined with a high‐fat diet (HFD) was employed to induce a DN mouse model. The observations in the mice model were consistent with the findings in DN patients. First, the expression of evf‐2 in the glomeruli showed a positive correlation with the urinary albumin/creatinine ratio (uACR) in the DN mouse model (STZ, Figure 1c–e). Second, there was a significant increase in evf‐2 expression, inflammatory factors, the number of positive PCNA staining, and a decrease in podocyte marker proteins in the glomeruli of DN mice (Figure 1f,g). B7‐1, Mcp‐1, or injury factor Claudin‐1 exhibited more colocalizations with podocin or SYNPO in DN mice compared with control mice, indicating podocyte injury and inflammation in the DN mouse model (Figure 1g). The expressions of podocyte marker proteins and inflammatory or injury‐related factors were further confirmed by western blotting (Figure 1h). The quantitative analysis of Figure 1f–h is shown in Figure S3 (Supporting Information). These results suggest that in the DN mouse model, cell cycle re‐entry and inflammation in podocytes may be the main contributors to albuminuria, closely associated with the increased expression of evf‐2.

### 2.2 Podocyte‐Specific Knockdown (KD) of Evf‐2 Alleviates Podocyte Injury, Inflammation, and Proteinuria in STZ Combined with HFD Treated Mice

**Figure 2:**
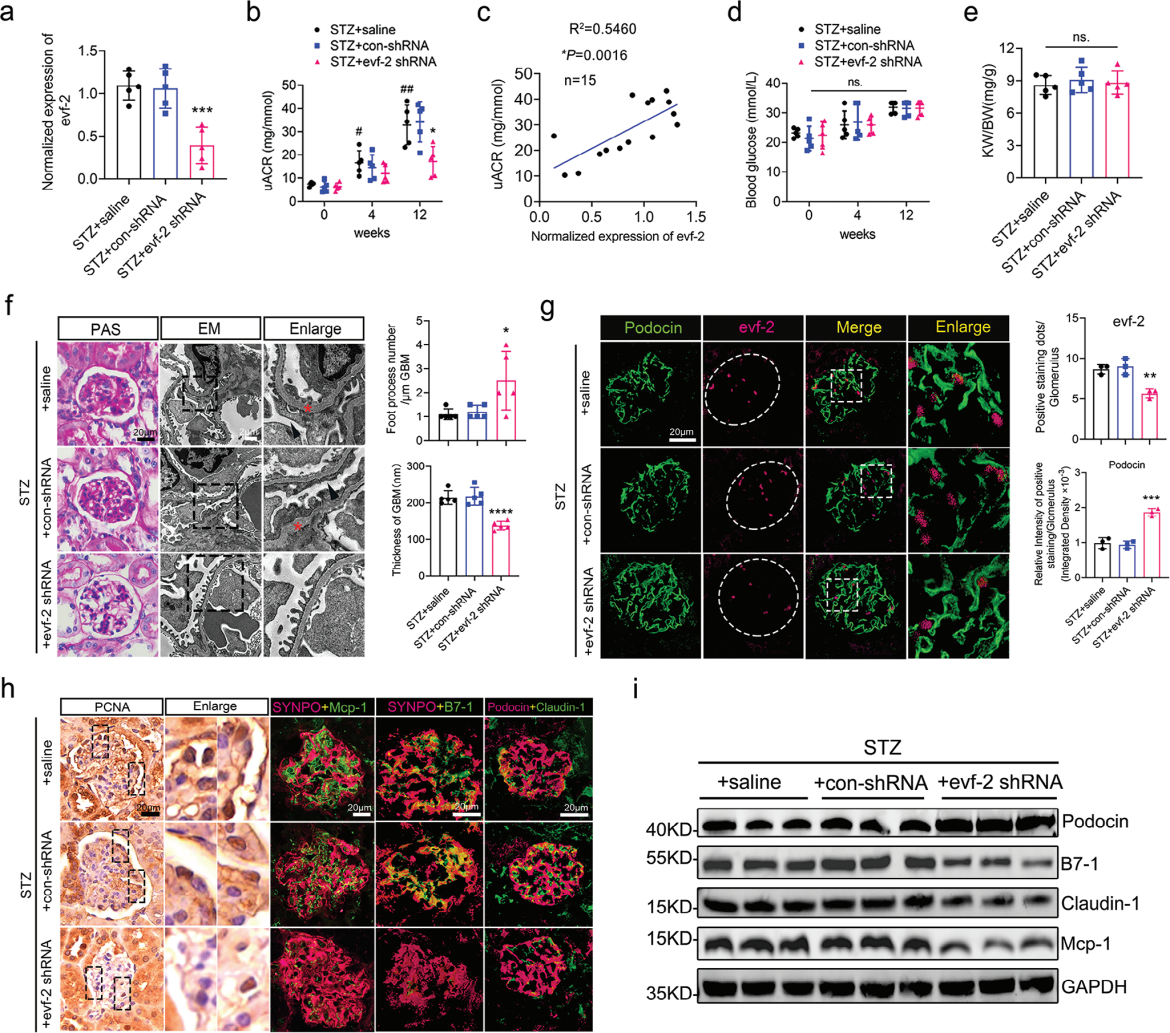
Podocyte‐specific knockdown of evf‐2 alleviates podocyte injury, inflammation, and proteinuria in STZ combined with HFD‐treated mice. a) The mice were categorized into three groups: saline treatment (STZ + saline), blank lentivirus treatment (STZ + con‐shRNA), and evf‐2 shRNA lentivirus treatment (STZ + evf‐2 shRNA). qRT‐PCR assays showing evf‐2 expression in isolated podocytes from three groups. n = 5 mice/group. b) uACR was assessed at various time points post‐injection for comparative analysis among the three groups. *p < 0.05, STZ + evf‐2 shRNA versus STZ + saline, #*p* < 0.05, ## < 0.01 (different time point in STZ + saline group), (two‐way ANOVA plus Dunnett's multiple comparisons test). n = 5 mice/group. c) A positive correlation was observed between uACR and evf‐2 expression levels in renal tissues. d and e) Random blood glucose (RBG) and kidney weight/body weight (KW/BW) ratio were compared among the three groups. n = 5 mice/group. f) Kidney tissues from mice treated with evf‐2 shRNA lentivirus were examined using Periodic Acid‐Schiff (PAS) staining and EM scanning after 12 weeks. Statistical analyses demonstrated significant changes in the number of foot processes per micrometer glomerular basement membrane (GBM) and basement membrane thickness in STZ‐induced mice under different treatments. Bar = 20 µm. Red stars indicated thickened basement membrane; black triangles indicated podocyte foot process effacement. Five observation fields were collected per sample. g) FISH analysis in paraffin‐embedded kidney tissue samples revealed a reduction in evf‐2 expression (magenta dots) and an increase in Podocin expression (green) in STZ + evf‐2 shRNA group mice. The intensity of the staining was quantified and compared in different samples (n = 3). Bar = 20 µm. h) Immunohistochemistry staining for PCNA among the three groups. Immunofluorescence analysis was conducted on OCT‐embedded frozen kidney tissues, staining for Podocin (magenta), SYNPO (magenta), Claudin‐1 (green), B7‐1 (green), and Mcp‐1 (green). Bar = 20 µm. i) Western blots were performed on isolated glomeruli from mouse kidneys, assessing the expressions of Podocin, B7‐1, Claudin‐1, and Mcp‐1. GAPDH was used as the loading control. *p < 0.05, ** < 0.01, *** < 0.001, **** < 0.0001, STZ + evf‐2 shRNA versus STZ + saline, (one‐way ANOVA plus Tukey's multiple comparisons test).

To further substantiate the role of evf‐2 in podocyte injury under DN conditions, we investigated the effects of podocyte‐specific KD of evf‐2 on podocyte injury, inflammation, and albuminuria in STZ mice. After a 4‐week HFD treatment, mice were subjected to saline (STZ + saline), blank lentivirus (STZ + con‐shRNA), or podocyte‐specific evf‐2 shRNA lentivirus (STZ + evf‐2 shRNA). The reduction in evf‐2 expression was confirmed by qRT‐PCR in isolated primary podocytes at 12 weeks after injection (**Figure** **2**a). Mice with evf‐2 KD in podocytes exhibited significantly lower uACR levels after 12 weeks compared with the other groups (Figure 2b). A positive correlation was observed between uACR and evf‐2 expression levels in renal tissues (Figure 2c). However, no changes were detected in random blood glucose (RBG) levels or kidney weight/body weight (KW/BW) ratios (Figure 2d,e). Evaluation of podocyte morphology, using PAS staining and EM, revealed an increase in foot process number and a decrease in the thickness of the glomerular basement membrane (GBM), indicating significantly reduced podocyte injury in the evf‐2 KD mice (Figure 2f). Notably, podocin expression gradually increased, accompanied by a concomitant decrease in evf‐2 expression in glomeruli (Figure 2g). The co‐localization of inflammatory or injury‐associated factors with podocyte marker proteins SYNPO or podocin was reduced in the evf‐2 KD mice glomerulus (Figure 2h, indicated by yellow color), suggesting reduced inflammation and injury in podocytes. Western blotting analysis of isolated glomeruli further confirmed these findings (Figure 2i). The number of podocytes engaged in cell cycle re‐entry was also decreased, as indicated by decreased positive staining for PCNA (Figure 2h). The quantitative analysis of Figure 2h,i is shown in the Figure S4 (Supporting Information). Taken together, these results suggest that inhibiting evf‐2 expression in kidney podocytes could represent an effective approach to alleviate podocyte injury, inflammation, and albuminuria associated with DN.

### 2.3 Podocyte‐Specific Overexpression of Evf‐2 Promotes Podocyte Injury, Inflammation, and Cell Cycle Re‐entry in Control Mice

To investigate whether the overexpression of evf‐2 could induce podocyte injury, inflammation, and cell cycle re‐entry, 10‐week‐old C57 mice (control mice) were injected with saline (CON+saline), blank lentivirus (CON+con‐LV), or podocyte‐specific evf‐2 overexpressed lentivirus (CON+evf‐2, containing the exon 3 of evf‐2) for 12 weeks.

**Figure 3:**
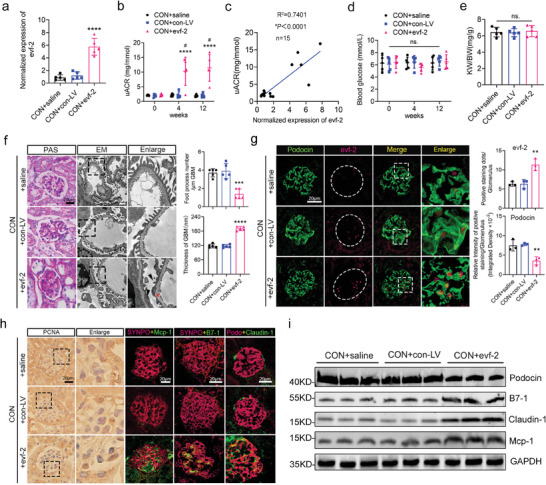
Podocyte‐specific overexpression of evf‐2 promotes podocyte injury, inflammation, and cell cycle re‐entry in control mice. a) Control mice were injected with saline (CON + saline), blank lentivirus (CON + con‐LV), or podocyte‐specific evf‐2 overexpression lentivirus (CON + evf‐2). qRT‐PCR assays showing evf‐2 expression in isolated podocytes from the three groups of mice. n = 5 mice/group. b) uACR was compared among the three groups at different times after injection. ****p < 0.0001, CON + evf‐2 versus CON + saline, #*p* < 0.05 (different time points in CON + evf‐2 group), (two‐way ANOVA plus Dunnett's multiple comparisons test). n = 5 mice/group. c) Positive correlation between uACR and evf‐2 expression levels in renal tissues. d and e) RBG and KW/BW were compared among the three groups. n = 5 mice/group. f) PAS staining and EM scanning analysis of podocyte injury in kidney tissues after 12 weeks of treatment with evf‐2 overexpression lentivirus. Bar = 20 µm. Red stars indicated thickened basement membrane; black triangles indicated podocyte foot process effacement. The statistical graphs depict the number of foot processes per micrometer GBM and the basement membrane thickness in control mice subjected to different treatments. Five observation fields were collected per sample. g) FISH analysis in paraffin‐embedded kidney tissue samples revealed an upregulation of evf‐2 expression in podocytes (magenta dots) and a concurrent decrease in Podocin (green) expression in the CON + evf‐2 group mice. Quantification of staining intensity demonstrated statistical significance (n = 3). Bar = 20 µm. h) Immunohistochemistry staining for PCNA exhibited evidence of podocyte cell cycle re‐entry in the glomeruli of mice in the CON + evf‐2 group. Immunofluorescence staining of Podocin (magenta), SYNPO (magenta), Mcp‐1 (green), B7‐1 (green), and Claudin‐1 (green) in OCT‐embedded frozen kidney tissues demonstrated varying intensities across different samples. Bar = 20 µm. i) Western blots of Podocin, B7‐1, Claudin‐1, Mcp‐1 in isolated glomeruli from mouse kidneys were conducted, with GAPDH as the loading control. *p < 0.05, ** < 0.01, *** < 0.001, **** < 0.0001, CON + evf‐2 versus CON + saline, (one‐way ANOVA plus Tukey's multiple comparisons test).

We observed a significant increase in uACR at 12 weeks post‐injection in the CON+evf‐2 group, and a positive correlation was noted (**Figure** **3**a–c). However, no remarkable changes in RBG or KW/BW (Figure 3d,e). Additionally, compared to the other two groups, mice in the CON+evf‐2 group exhibited increased renal tissue matrix, a thickened basement membrane (indicated by red stars), and podocyte foot process effacement (indicated by black triangles) as revealed by PAS staining and EM scanning (Figure 3f). The overexpression of evf‐2 in CON+evf‐2 group mice was further confirmed by fluorescence in situ hybridization (FISH) (Figure 3g). As evf‐2 expression gradually increased in the mouse glomeruli, the expression of podocyte marker proteins gradually decreased, while the expression of inflammatory and injury‐associated factors increased in these podocytes (Figure 3g–i). The number of podocytes engaged in cell cycle re‐entry was also increased, as indicated by positive staining for PCNA (Figure 3h). The quantitative analysis of Figure 3h,i is shown in Figure S5 (Supporting Information). These results demonstrate that increased evf‐2 expression promotes podocyte injury, cell cycle re‐entry, inflammation, and albuminuria in C57 mice.

### 2.4 Podocyte‐Specific Evf‐2 Knockout Remarkably Reduces Podocyte Injury and Inflammation in STZ‐Induced Diabetic Mice

To further elucidate the role of evf‐2 in podocytes, we generated podocyte‐specific evf‐2 knockout (evf‐2 KO) mice, as illustrated in Figure S6a and b (Supporting Information). Notably, no significant differences in RBG, KW/BW, and uACR were observed between flox/flox mice and evf‐2 KO mice (Figure S6c, Supporting Information). To confirm that podocyte‐specific knockout of evf‐2 can alleviate podocyte injury and inflammation in DN, we employed 6‐week‐old male evf‐2 KO and flox/flox mice (as controls) to establish DN models through STZ combined with an HFD induction. The construction strategy and specimen collection are detailed in Figure S6d (Supporting Information).

**Figure 4:**
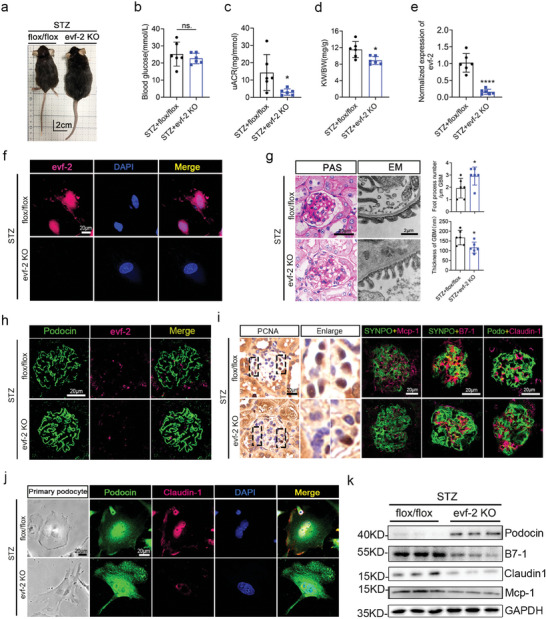
Podocyte‐specific evf‐2 knockout alleviates podocyte injury and inflammation in STZ‐induced DN mice model. a) Visual comparison of the appearance of control (flox/flox) and podocyte‐specific evf‐2 knockout (evf‐2 KO) mice after STZ induction. b–d) Comparative analysis of RBG(b), uACR(c) and KW/BW(d) levels between the two groups of mice after 7 weeks of STZ injection. n = 6 mice/group. e) qRT‐PCR used to detect evf‐2 expression in primary cultured podocytes isolated from the glomeruli of evf‐2 KO and flox/flox mice after STZ induction. n = 6 mice/group. f) FISH on primary podocytes isolated from STZ‐treated evf‐2 KO mice and STZ‐treated control mice. Bar = 20 µm. g) PAS staining and EM scanning of kidney tissues revealing reduced damage in the kidney structures of the evf‐2‐KO mice following STZ treatment. The statistical chart compares the number of podocyte foot processes per micrometer GBM and the thickness of basement membrane between the two groups. Bar = 20 µm. h) FISH showing almost no expression of evf‐2 in the KO mouse glomeruli. Bar = 20 µm. The intensity of the staining is quantified and compared in different samples (n = 3). i) Positive staining for PCNA decreased in the STZ+evf‐2 KO group mice. Immunofluorescence analyses of Podocin (green), SYNPO (green), Claudin‐1 (magenta), B7‐1 (magenta), Mcp‐1 (magenta) in OCT‐embedded frozen kidney tissues. The intensity of the staining is quantified and compared in different samples (n = 3). Bar = 20 µm. j) Bright light observations and immunofluorescence analyses of Claudin‐1 (magenta) and Podocin (green) in primary isolated podocytes of the glomeruli of evf‐2‐KO and flox/flox mice after STZ induction. Bar = 20 µm. k) Western blotting of Podocin, B7‐1, Claudin‐1, Mcp‐1 in primary isolated podocytes from two groups after STZ injection. GAPDH was used as the loading control. *p < 0.05, ** < 0.01, *** < 0.001, **** < 0.0001, (unpaired t‐test).

After 7 weeks of STZ injection, a visual inspection revealed significant differences in the appearance of the two groups, with flox/flox mice displaying smaller, thinner, and poorer conditions compared to evf‐2 KO mice (**Figure** **4**a). RBG levels increased significantly in both groups after STZ injection, with no notable difference between the two groups (Figure 4b). KW/BW and uACR in evf‐2 KO mice were significantly lower than those in flox/flox mice at 7 weeks after STZ injection (Figure 4c,d). The knockout of evf‐2 in primary cultured podocytes isolated from the glomeruli was validated through qRT‐PCR (Figure 4e). FISH analysis also validated the knockout of evf‐2 and confirmed the main localization of evf‐2 in the nuclear and nuclear‐related areas (Figure 4f). Matrix accumulation and podocyte foot process effacement, evident in PAS staining and EM, were notably alleviated in evf‐2 KO mice under STZ treatment (Figure 4g), indicating a protective role of evf‐2 KO. This protective effect was further supported by FISH and immunofluorescence results of the glomerulus (Figure 4h,i). Podocin and SYNPO expression in STZ+flox/flox mice exhibited green fluorescence fragmentation, indicating severe podocyte injury, while evf‐2 KO mice showed milder damage. Positive staining for PCNA was also decreased in the STZ+evf‐2 KO mice compared with the STZ+flox/flox mice (Figure 4i). Besides, upregulated Claudin‐1 expression, and downregulated podocin expression, were also observed in primary isolated podocytes from both groups of mice (Figure 4j). Expression of the podocyte injury protein Claudin‐1 and the inflammatory factors B7‐1, Mcp‐1 in primary isolated podocytes of STZ+flox/flox mice was significantly higher than in STZ+evf‐2 KO mice (Figure 4k). The quantitative analysis of Figure 4h–k is shown in Figure S6e–g (Supporting Information). All these experimental findings collectively indicate that podocyte‐specific knockout of evf‐2 can alleviate proteinuria, inflammation, and podocyte injury in a diabetic mice model.

### 2.5 Podocyte‐Specific Evf‐2 Knockout Significantly Attenuates Podocyte Injury and Inflammation in LPS‐Induced Mouse Model

**Figure 5:**
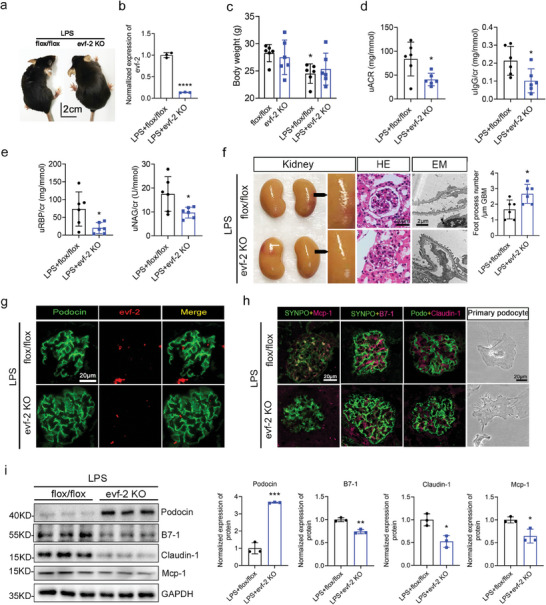
Podocyte‐specific knockout of evf‐2 alleviates podocyte injury and inflammation in LPS‐induced mouse model. a) The visual appearance of flox/flox and KO mice after LPS induction. b) qRT‐PCR was employed to detect evf‐2 expression in primary isolated podocytes of the glomeruli of KO or flox/flox mice after LPS induction. n = 3 mice/group. c) The body weight of flox/flox and KO mice after LPS induction. *p < 0.05, LPS + flox/flox versus flox/flox, (two‐way ANOVA plus Sidak's multiple comparisons test). n = 6 mice/group. d and e) Indicators of glomerular injury (d), including uACR and urinary immunoglobulin G/creatinine ratio (uIgG/cr), and indicators of tubular injury (e), such as urinary N‐acetyl‐β‐D‐glucosaminidase/creatinine ratio (uNAG/cr) and urinary retinol‐binding protein/creatinine ratio (uRBP/cr), were analyzed in the KO mice. n = 6 mice/group. f) Kidney images of flox/flox and KO mice after LPS induction. Hematoxylin‐eosin staining (HE) and EM analysis of kidney tissues. The statistical chart compares the number of podocyte foot processes per micrometer GBM between the two groups. Bar = 20 µm. g) FISH analysis of the KO mice glomeruli under LPS treatment. Bar = 20 µm. h) Immunofluorescence analyses of SYNPO (green), Podocin (green), Claudin‐1 (magenta), B7‐1 (magenta), Mcp‐1 (magenta) in OCT‐embedded frozen kidney tissues. Bright light observations of primary isolated podocytes. Bar = 20 µm. i) Western blotting of Podocin, B7‐1, Claudin‐1, Mcp‐1 in primary isolated podocytes. Quantification of the western blot data is shown in the right panels. Band intensities were analyzed in ImageJ and normalized to that of GAPDH. *p < 0.05, ** < 0.01, *** < 0.001, **** < 0.0001, (unpaired t‐test).

In this study, we utilized the DN model to investigate the impact of evf‐2 and discovered its regulatory role in the expression of inflammatory factors. While inflammation is acknowledged as a significant contributor to DN development, it is essential to note that DN is not a classic inflammatory disease, as outlined in numerous publications.^[^ ^8^, ^10^ ^]^ To further substantiate the regulatory role of evf‐2 in inflammation, we employed an LPS‐induced mouse model using podocyte‐specific evf‐2 KO mice. Remarkable differences in appearance were observed between control (flox/flox) and KO mice after LPS induction (**Figure** **5**a). Flox/flox mice exhibited more pronounced weight loss, coarse hair, and shortness of breath compared with evf‐2 KO mice under LPS treatment (Figure 5c). The knockout of evf‐2 in primary cultured podocytes isolated from the glomeruli was confirmed through qRT‐PCR (Figure 5b). We employed uACR and urinary immunoglobulin G/creatinine ratio (uIgG/cr) as indicators of glomerular injury (Figure 5d), and urinary N‐acetyl‐β‐D‐glucosaminidase/creatinine ratio (uNAG/cr) and urinary retinol‐binding protein/creatinine ratio (uRBP/cr) as indicators of tubular injury (Figure 5e).^[^ ^23^ ^]^ Under LPS treatment, both glomerular and tubular injury indicators were significantly lower in evf‐2 KO mice compared to flox/flox mice, indicating a more severe injury in LPS‐induced flox/flox mice.

Moreover, following LPS treatment, flox/flox mice exhibited a noticeable granular appearance on the kidney surface, while evf‐2 KO mice displayed a relatively smoother surface. Morphological characteristics were more severely disrupted in flox/flox mice, with a significant foot process effacement of podocytes compared to evf‐2 KO mice (Figure 5f). Compared with LPS‐induced evf‐2 KO mice, LPS‐induced flox/flox mice showed less expression of podocin in the glomeruli (Figure 5g), and more expression of inflammatory factors and podocyte injury factors (Figure 5h). Bright light observations of the primarily isolated podocytes revealed a significant reduction in podocyte foot processes. Synaptic links between cells were disrupted (Figure 5h). The quantitative analysis of Figure 5g,h is shown in Figure S7 (Supporting Information). This was corroborated by immunoblotting of primary cultured podocytes isolated from the glomeruli (Figure 5i). Collectively, these results suggest that podocyte‐specific evf‐2 knockout significantly attenuates LPS‐induced acute inflammatory injury.

### 2.6 Evf‐2 Upregulation Induces Podocyte Injury via Cell Cycle Re‐entry and Inflammation

**Figure 6:**
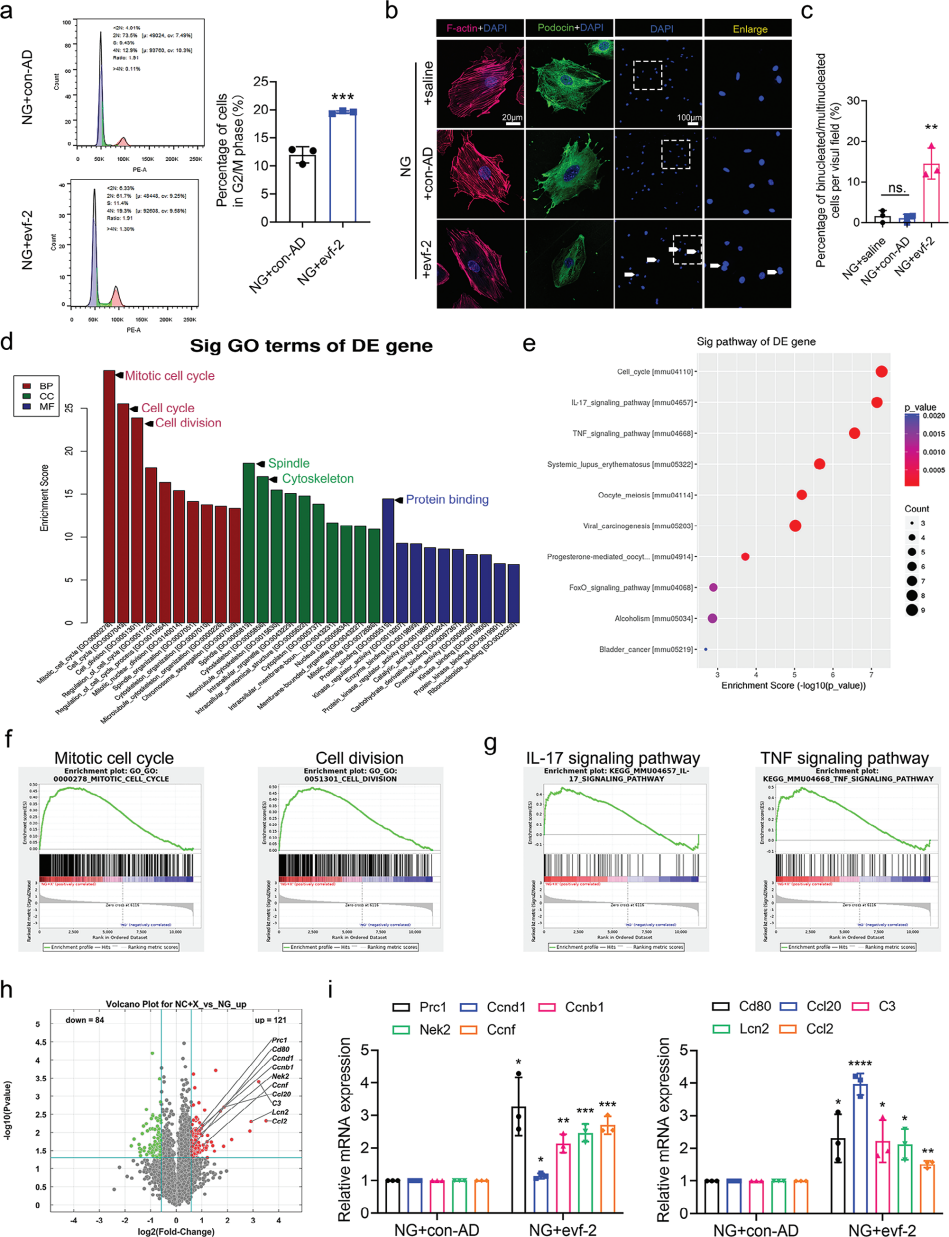
Overexpression of evf‐2 induces podocyte injury through cell cycle re‐entry and inflammation in vitro. a) Flow cytometry analysis of the cell cycle in the cultured mouse podocyte cell line (MPC5) under blank adenovirus (NG+con‐AD) and evf‐2 overexpression conditions (NG+evf‐2). The percentage of cells in the G2/M phase is presented in the right panel (n = 3). ***p < 0.001, (unpaired t‐test). b) Rhodamine phalloidin labeling revealed cytoskeletal damage in MPC cells in the NG group (5.6 mM), NG+con‐AD group, and NG+evf‐2 group. Representative photomicrographs of immunofluorescence staining for Podocin (green) and binucleation (DAPI) of podocytes. An enlarged section (dashed rectangle) is presented, white arrow indicates binucleated podocytes. Bar = 20 µm. c) The percentage of binucleated/multinucleated cells per visual field is presented (n = 3). **p < 0.01, (one‐way ANOVA plus Tukey's multiple comparisons test). d) Gene Ontology (GO) analysis of differentially expressed (DE) genes from RNA‐seq results performed on MPCs. e) Pathway analysis obtained for the enrichment score dot plot, showcasing the top ten pathways. f) Gene Set Enrichment Analysis (GSEA) of biological processes, including mitotic cell cycle and cell division. g) GSEA of Kyoto Encyclopedia of Genes and Genomes (KEGG) pathway, focusing on IL‐17 and TNF signaling pathway. h) A volcano plot depicting differential expression of mRNAs. The top five up‐regulated mRNAs in cell cycle and inflammatory biological processes were selected, respectively. i) qRT‐PCR was utilized to verify the expression of selected mRNAs. Each experiment was repeated three times. *p < 0.05, ** < 0.01, *** < 0.001, **** < 0.0001, (unpaired t‐test).

We have successfully demonstrated the detrimental impact of evf‐2 upregulation on podocytes. Moreover, knockdown or knockout of evf‐2 has proven effective in alleviating podocyte damage and inflammation in cases of diabetic nephropathy or LPS‐induced podocyte injury. Despite these positive outcomes, the underlying mechanism remains unclear. Interestingly, binucleated podocytes (Figure 1b) and increased PCNA staining, indicative of cellular proliferation, were observed in glomeruli of DN patients' biopsies, STZ‐induced mice, and mice treated with podocyte‐specific overexpression of evf‐2 (Figures 1a,g and 3h). These results suggest that podocytes have cell cycle disorders and inflammation in diabetic nephropathy. Podocytes, as terminally differentiated cells, typically reside in the G0 phase. However, flow analysis revealed an increased percentage (NG+con‐AD:12.9%; NG+evf‐2:19.3%, *p* < 0.001) of podocytes in the G2/M phase following evf‐2 overexpression (**Figure** **6**a).

Additionally, immunofluorescence analysis revealed that evf‐2 overexpression resulted in a decrease in the podocyte marker protein podocin and disruption of the podocyte cytoskeleton, concomitant with an augmentation in the proportion of binucleated podocytes (Figure 6b,c, indicated by white arrows). Based on these observations, we postulated that the upregulation of evf‐2 under DN conditions induces podocyte injury through cell cycle re‐entry and inflammation.

To validate this hypothesis, we conducted RNA sequencing on podocytes overexpressing evf‐2 and those without. The results revealed a significant upregulation of mRNAs in the NG+evf‐2 group compared to the NG+con‐AD group, particularly in the categories of chemokines, cytokines, and cell cycle‐related genes. Both GO analysis and KEGG pathway analysis highlighted the cell cycle as the most enriched term among upregulated mRNAs (Figure 6d,e). IL‐17 and TNF signaling pathways ranked second and third in KEGG analysis (Figure 6e), a conclusion further supported by Gene Set Enrichment Analysis (GSEA) of biological processes (Figure 6f,g). To further corroborate these findings, qRT‐PCR was performed on the top five upregulated mRNAs related to the cell cycle and inflammation, as illustrated in the volcano plot of RNA‐sequencing results (Figure 6h,i). Collectively, these experiments strongly indicate that upregulation of evf‐2 induces cell cycle re‐entry and inflammation in podocytes.

### 2.7 Evf‐2 Binds to Proteins Involved in mRNA Processing or Alternative Splicing to Induce Podocyte Cell Cycle Re‐entry or Inflammation

**Figure 7:**
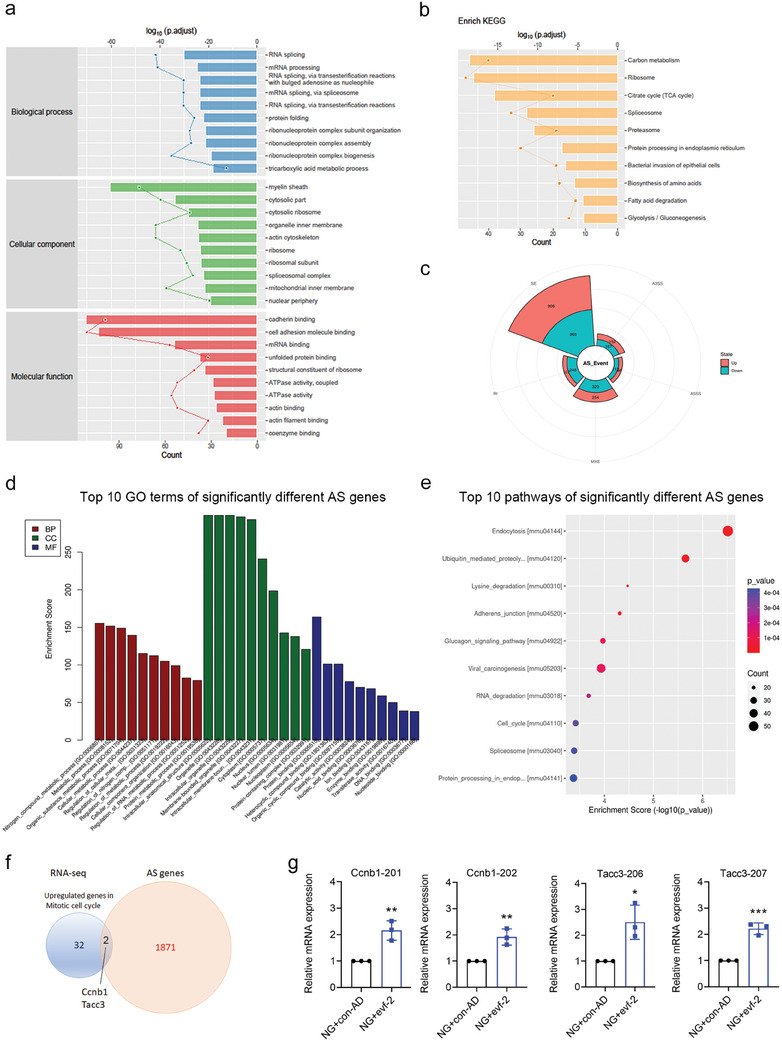
Evf‐2 binds to proteins involved in mRNA processing or alternative splicing to induce podocyte cell cycle re‐entry or inflammation. a) GO and KEGG b) analyses of the RNA binding proteins of lncRNA evf‐2. c) Alternative splicing (AS) events analysis of RNA sequencing results performed on MPCs overexpressing evf‐2. d,e) The top 10 terms of GO (d) or KEGG (e) result of significantly different AS genes. f) The Venn result of significantly upregulated genes of RNA‐seq in mitotic cell cycle and significantly different AS genes. g) qRT‐PCR of splicing variants of Ccnb1 and Tacc3. *p < 0.05, ** < 0.01, *** < 0.001, (unpaired t‐test).

Our earlier findings indicated that evf‐2 is localized in the nucleus and the perinuclear region (Figure 4f), prompting us to hypothesize that evf‐2 regulates downstream gene expression by binding to specific proteins. To elucidate the underlying mechanism, we conducted ChIRP‐MS analysis on podocytes overexpressing evf‐2 to identify its binding proteins. The results revealed that the majority of binding proteins were enriched in mRNA processing, RNA splicing, protein folding, and the ribonucleoprotein complex (**Figure** **7**a,b). This suggests that evf‐2 may modulate downstream gene expression by binding to proteins associated with the ribonucleoprotein complex, potentially affecting downstream gene expression through alternative splicing or processes related to RNA metabolism. To further validate this hypothesis, additional analysis of RNA sequencing results revealed significant alternative splicing events within cells following evf‐2 overexpression (Figure 7c).

Subsequent GO and KEGG analyses of mRNAs exhibiting significant alternative splicing revealed notable alternations in processes related to cellular metabolism, RNA metabolism, and the cell cycle (Figure 7d,e). However, no significant alternative splicing events were observed in mRNA encoding inflammation factors. To further elucidate which genes' alternative splicing may be involved in the observed cell cycle changes following evf‐2 overexpression, we identified two significantly upregulated genes, Ccnb1 and Tacc3, showing significant alternative splicing events (Figure 7f). Both genes exhibited two splicing isoforms, Ccnb1‐201/202 and Tacc3‐206/207, consistent with the sequencing results validated by qRT‐PCR (Figure 7g). These findings suggest that evf‐2 may regulate transcription, alternative splicing, and RNA stability by interacting with proteins associated with the ribonucleoprotein complex or other proteins, thereby participating in the expression of cell cycle and inflammation‐related genes. However, the mechanisms underlying the regulation of mRNA expression for cell cycle‐associated proteins and inflammatory factors appear to differ.

### 2.8 Knockdown of hnRNPU Alleviates the Cell Cycle Re‐entry and Increased Expression of Inflammatory Factors Induced by Evf‐2 Overexpression

**Figure 8:**
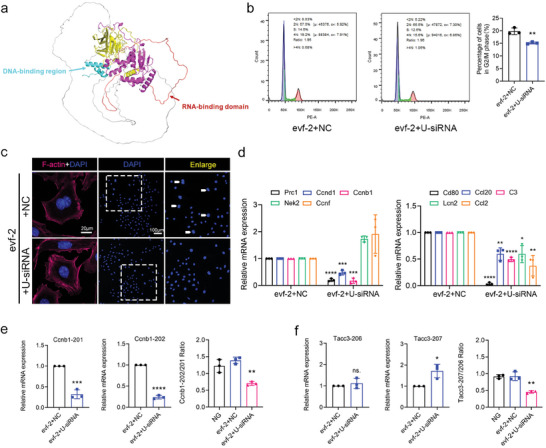
Knockdown of hnRNPU alleviates the cell cycle re‐entry and increased expression of inflammatory factors induced by evf‐2 overexpression. a) The predicted structure of hnRNPU protein by AlphaFold. This predicted hnRNPU protein consists of four functional regions: DNA binding region (cyan), ATP binding region (purple), RNA binding region (red), and the unknown functional region (yellow). b) Flow cytometry analysis demonstrated knockdown of hnRNPU partially reversed podocytes entering the G2/M phase in evf‐2 overexpressed podocytes. The quantitative analysis is shown in the right panel. c) Representative photomicrographs of rhodamine phalloidin staining and binucleation (DAPI) of podocytes in the evf‐2 + U‐siRNA (transfected with hnRNPU siRNA) and evf‐2 + NC group (transfected with siRNA NC). An enlarged section (dashed rectangle) is presented, white arrow indicates binucleated podocytes. Bar = 20 µm. d) qRT‐PCR analysis of cell cycle and inflammation‐related mRNAs indicated in Figure 6i. in podocytes overexpressing evf‐2 with or without knockdown of hnRNPU (evf‐2+siRNA NC or evf‐2+U‐siRNA). e and f) qRT‐PCR of two splicing isomers of Ccnb1 (e) and Tacc3 (f) and their respective ratios under different treatments. *p < 0.05, ** < 0.01, *** < 0.001, **** < 0.0001, (unpaired t‐test/one‐way ANOVA plus Tukey's multiple comparisons test).

It is noteworthy that among the ribonucleoproteins identified in the ChIRP‐MS results, heterogeneous nuclear ribonucleoprotein U (hnRNPU) emerged as the most prominent spliceosome‐associated protein bound to evf‐2. hnRNPU is known to interact with RNA, DNA, and proteins, thereby influencing their expression.^[^ ^20^ ^]^ AlphaFold prediction revealed the structure of hnRNPU protein, highlighting both DNA and RNA binding domains (**Figure** **8**a). Interestingly, the overexpression of evf‐2 did not affect the expression of hnRNPU at the mRNA or protein level (Figure S8a and b, Supporting Information).

To further investigate the role of hnRNPU in podocytes overexpressing evf‐2, we transfected hnRNPU‐siRNA into these cells. The effectiveness of evf‐2 overexpression and hnRNPU siRNA knockdown was demonstrated in Figure S8c–e (Supporting Information). Knockdown of hnRNPU in podocytes overexpressing evf‐2 led to a decreased number of cells entering the G2/M phase (evf‐2+NC:19.2%; evf‐2+U‐siRNA:15.6%, *p*<0.01) and a reduction in the proportion of binucleated cells (Figure 8b,c). Additionally, the disorganization of the podocyte cytoskeleton improved in the evf‐2 + hnRNPU‐siRNA group compared to the evf‐2 + NC group (Figure 8c). The quantitative analysis of binucleated podocytes is shown in Figure S8f (Supporting Information). Furthermore, the knockdown of hnRNPU partially reversed the upregulation of the cell cycle and inflammation‐related mRNA expression (Figure 8d), except for Nek2 and Ccnf. After hnRNPU‐specific knockdown in cultured podocytes, the expression of Ccnb1‐201 and Ccnb1‐202 were decreased significantly, but not Tacc3 splicing variants. However, the proportions of splicing variants of both Ccnb1 and Tacc3 changed after hnRNPU knockdown (Figure 8e,f). These results suggest that hnRNPU mediates podocyte cell cycle re‐entry and upregulated expressions of inflammatory factors induced by evf‐2 overexpression. Additionally, hnRNPU primarily regulates the expression of inflammatory factors without affecting their alternative splicing. Concerning cell cycle‐related genes, hnRNPU can influence both the expression and splicing of Ccnb1, but only impacts the alternative splicing of Tacc3.

### 2.9 hnRNPU Directly Binds to the DNA or RNA of Cell Cycle‐Related Proteins and Chemokines to Regulate their Expression

**Figure 9:**
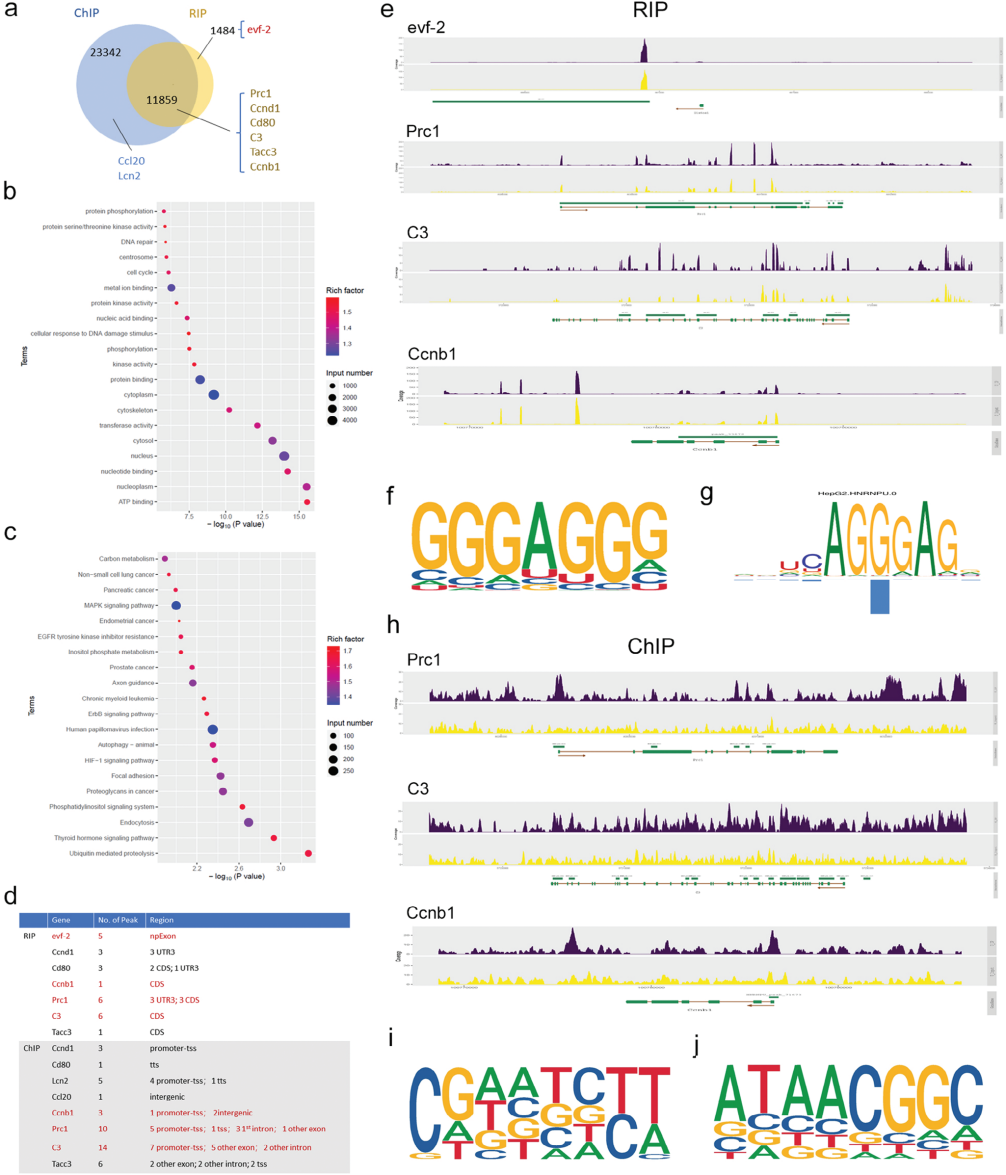
HnRNPU directly binds to cell cycle‐related genes and inflammation factors through RNA and/or DNA binding sites. a) The Venn diagram depicts the results of ChIP‐sequencing and RIP‐sequencing conducted on podocytes overexpressing evf‐2 by using hnRNPU antibody. The most significantly upregulated genes in the cell cycle or inflammation, as selected from Figure 6h, are presented. b and c) GO (b) and KEGG pathway analysis (c) is performed on genes overlapping of ChIP and RIP assays. d) The binding regions and peaks of genes binding with hnRNPU in ChIP or RIP assays indicated in panel a. e) The gene plot results from RIP assays illustrate the genes binding with hnRNPU. f,g) The most probable specific motif sequence of RNA binding with hnRNPU was analyzed based on the results (f), revealing similarity to the binding motif predicted by mCrossBase (g). h) The gene plot results from ChIP assays illustrate the genes binding with hnRNPU. i, j) The top two binding motifs of target DNA.

To identify the genes or mRNAs that can bind to hnRNPU, we conducted chromatin immunoprecipitation sequencing (ChIP‐seq) and RNA immunoprecipitation sequencing (RIP‐seq) on podocytes overexpressing evf‐2. The experimental results revealed that hnRNPU can bind to evf‐2, as well as simultaneously bind to both the DNA and RNA of various cell cycle proteins and inflammatory factors. For individual inflammatory factors, such as Lcn2 and Ccl20, hnRNPU only binds to their DNA (**Figure** **9**a).

The GO and KEGG pathway analyses (Figure 9b,c) provided valuable insights into the overlapping genes identified in both ChIP and RIP analyses, highlighting their crucial regulatory roles in cell cycle and inflammation‐related signaling pathways. Further analysis elucidated the precise binding regions and peaks of hnRNPU with genes associated with cell cycle or inflammation (Figure 9d). We found that hnRNPU can bind to the transcription start site (TSS), exon, and intron regions of DNA, as well as to the 3′‐untranslated region (3′‐UTR) and coding sequence (CDS) of RNA, laying the foundation for further elucidating hnRNPU's mechanism of action. The gene plots depicted several typical factors bound by hnRNPU in RIP results (Figure 9e), particularly emphasizing evf‐2, Prc1, C3, and Ccnb1 as representative genes bound to hnRNPU. The most probable specific motif sequence of RNA binding with hnRNPU was analyzed based on the results (Figure 9f), revealing similarity to the binding motif predicted by mCrossBase (Figure 9g).^[^ ^24^ ^]^ Selective gene plots from the ChIP assay are presented in Figure 9h, showcasing the top two binding motifs of target DNA in Figure 9i,j. qRT‐PCR was employed to confirm the results of the binding genes identified in RIP and ChIP assays (Figure S9a and b, Supporting Information). The detailed calculation process is presented in the Supporting information.

These confirmatory results provide robust evidence supporting the direct association of hnRNPU with crucial genes regulating the cell cycle and inflammation, further elucidating hnRNPU's intricate role in these cellular processes.

## 3 Discussion

DN has become the leading cause of end‐stage renal disease, with the incidence and prevalence rising significantly each year, imposing a heavy social and economic burden worldwide. Podocyte injury, as a crucial mechanism in diabetic nephropathy, is the primary cause of proteinuria and has been a focal point and challenge in research.^[^ ^4^, ^5^, ^6^ ^]^ Our team has been dedicated to studying the mechanisms of DN and podocyte injury for many years.^[^ ^16^, ^25^, ^26^ ^]^

Under normal physiological conditions, podocytes maintain a highly differentiated state and remain in the interphase of the cell cycle. However, in pathological conditions, podocytes can progress into the mitotic phase and undergo nuclear division, which is considered a significant factor contributing to proteinuria, although the exact mechanisms remain unclear.^[^ ^27^ ^]^ The unique cytoskeletal structure of podocytes, characterized by foot processes, renders them unable to complete normal cytokinesis, ultimately resulting in podocyte injury, detachment, and death (known as mitotic catastrophe).^[^ ^28^ ^]^ Studies have shown that podocytes undergo mitosis in kidney diseases such as DN,^[^ ^29^ ^]^ focal segmental glomerulosclerosis (FSGS),^[^ ^30^ ^]^ and Alport syndrome.^[^ ^31^ ^]^ In this study, electron microscopy examinations of kidney tissues from DN patients revealed podocytes exhibiting binucleation or multinucleation, accompanied by increased positive immunohistochemical staining for PCNA, indicating that podocytes indeed undergo mitosis in DN.

Previous studies have indicated the significant role of lncRNAs in podocyte injury in DN. For instance, LncRNA ENST00000436340 promotes podocyte injury by facilitating the association of PTBP1 with RAB3B.^[^ ^32^ ^]^ Similarly, lncRNA MALAT1 promotes renal fibrosis by targeting the miR‐2355‐3p/IL6ST Axis.^[^ ^33^ ^]^ Additionally, LINC00355 mediates CTNNBIP1 promoter methylation and promotes endoplasmic reticulum stress‐induced podocyte injury.^[^ ^34^ ^]^

The lncRNA evf‐2 proposed in this study was selected based on our team's earlier research findings. Currently, research on lncRNA evf‐2 predominantly focuses on cancer, with most mechanisms being associated with the competing endogenous RNAs (ceRNA) mechanism.^[^ ^35^, ^36^ ^]^ For example, lncRNA evf‐2 enhances stemness in osteosarcoma by regulating the miR‐129‐5p/DLK1 axis,^[^ ^37^ ^]^ promotes breast cancer progression by regulating the miR‐505‐3p/RUNX2 axis,^[^ ^38^ ^]^ promotes cervical cancer progression by targeting the miR‐16‐5p/ARPP19 axis.^[^ ^39^ ^]^ Our investigation demonstrated an upregulation of lncRNA evf‐2 expression in renal biopsy specimens from DN patients, which correlated with podocyte mitosis and inflammation. Similar observations were made in a mouse model induced by STZ combined with HFD. To explore the role and mechanism of evf‐2, this study employed podocyte‐specific knockdown of evf‐2, podocyte‐specific overexpression of evf‐2, and podocyte‐specific knockout of evf‐2 in different mice separately. The results demonstrated that increased expression of evf‐2 leads to podocyte mitosis and inflammation, and podocyte‐specific knockout of evf‐2 alleviates podocyte injury induced by LPS or DN. RNA sequencing analysis revealed that the upregulation of evf‐2 expression significantly increased mRNA expression related to the cell cycle and inflammation in podocytes. Moreover, alternative splicing events were observed in cell cycle‐related genes but not in inflammation‐related factors. This underscores that the upregulation of evf‐2 expression can lead to podocyte injury by influencing the mRNA expression of cell cycle‐related proteins or inflammatory factors, as well as alternative splicing, albeit through different mechanisms.

**Figure 10:**
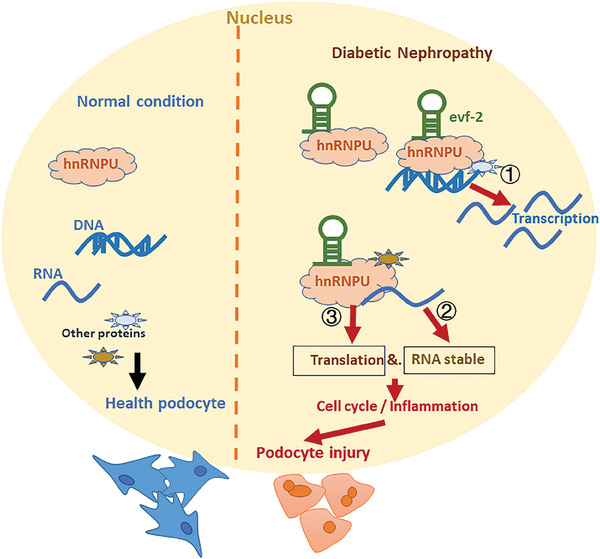
Proposed mechanism by which lncRNA evf‐2 induces podocyte injury in DN. Left panel: Under normal conditions, lncRNA evf‐2 expression in renal podocytes is minimal, while hnRNPU is expressed at a basal level in healthy podocytes. Right panel: In DN, lncRNA evf‐2 expression is upregulated, binding with hnRNPU, thereby leading to increased expression of cell cycle‐related genes and inflammatory factors, possibly through transcriptional activation, alternative splicing, or RNA stability and translation regulation.

In terms of mechanistic exploration, unlike most studies on ceRNA mechanisms, we primarily investigated how lncRNA evf‐2 regulates downstream gene expression by binding to specific RNA‐binding proteins, particularly by influencing alternative splicing of downstream genes. In our study, ChIRP‐MS revealed that evf‐2 binds to various heterogeneous nuclear ribonucleoproteins (hnRNPs) and participates in multiple biological processes such as RNA splicing and protein folding. Among hnRNPs, evf‐2 had a notable binding affinity to hnRNPU. hnRNPU is one type of RNA‐binding protein presented in the cell nucleus, which may also shuttle between the cytoplasm and nucleus under certain stimuli.^[^ ^20^ ^]^ It participates in gene transcription and subsequent post‐transcriptional regulation processes of newly synthesized RNA, as well as nucleic acid metabolism. The functions of hnRNPU are diverse and complex, involving pre‐mRNA packaging, selective splicing regulation, and chromatin remodeling.^[^ ^21^ ^]^ It is widely expressed in the human body and implicated in various processes such as renal tumors, very low‐density lipoprotein secretion, and adipocyte metabolism.^[^ ^40^, ^41^ ^]^ Additionally, hnRNPU plays a crucial role in mitosis. It co‐localizes with TPX2 and Aurora‐A as a spindle regulator at spindle poles and microtubules to control mitotic processes, chromosome alignment, and spindle assembly.^[^ ^21^ ^]^ However, there is limited research on its role in DN and podocyte injury. This study represents the first exploration of hnRNPU's role in podocyte injury associated with diabetic kidney injury. We found that the knockdown of hnRNPU in podocytes partially restored the upregulation of mRNAs related to cell cycle re‐entry and inflammation induced by evf‐2. Additionally, it altered the expression ratio of two splice isoforms of Ccnb1 (202/201) and Tacc3 (207/206), suggesting that hnRNPU can modulate the mRNA of cell cycle‐associated proteins through transcription and alternative splicing, without affecting the alternative splicing process of inflammatory factor mRNA. Through Alpha folding analysis of hnRNPU's protein conformation and integrating the results of ChIP and RIP experiments, it was confirmed that hnRNPU indeed bind separately to the DNA or RNA of cell cycle‐related proteins and inflammatory factors, potentially regulating transcription, alternative splicing, RNA stability, and protein translation processes (**Figure** **10**). However, the stability and translational regulation of RNA remains speculative based on this study, necessitating further experimental validation.

Previous studies have confirmed that in DN, upregulation of Ccnb1 expression and Myeloid‐derived growth factor deficiency can both lead to podocyte mitosis and subsequent death.^[^ ^29^, ^30^ ^]^ Our research also confirms the upregulation of lncRNA evf‐2 expression in podocytes of DN, which can lead to increased expression of Ccnb1, closely related to podocyte re‐entering into the cell cycle, consistent with previous studies. Furthermore, Tacc3, as an important regulator of the cell cycle, has been shown to play a significant role in the development of various tumors, such as pancreatic cancer, bladder cancer, and the centrosome amplification process in highly invasive breast cancer cells.^[^ ^42^ ^]^ Targeting Tacc3 has been demonstrated to induce immunogenic cell death in HER2‐positive breast cancer and enhance T‐DM1 response, making it a potential target for anti‐tumor drugs.^[^ ^43^ ^]^ However, its role in DN and podocyte injury has not been reported and warrants further exploration.

Additionally, alternative splicing is a crucial mechanism for maintaining biological diversity. Cells generate multiple mRNA transcripts through selective splicing to ensure diversity in the proteome. Splicing regulation mechanisms impact almost all signaling pathways. Aberrant alternative splicing is implicated in various human diseases, including cancer.^[^ ^44^ ^]^ Currently, there is limited research on alternative splicing in podocyte injury, particularly regarding the regulation of cell cycle‐related genes. Studies on the role of splice isoforms of Tacc3 and Ccnb1 in DN are lacking. This study proposes for the first time that splice isoforms of Ccnb1 or Tacc3 may be associated with podocyte mitosis. Database analysis suggests that Ccnb1‐201 contains multiple binding sites for CDK2 (Cyclin‐Dependent Kinase 2), which may promote re‐entry into the cell cycle. However, it was not testified by the experiment in this study. These findings warrant further investigation and represent a limitation of this study.

Indeed, the re‐entry of podocytes into the cell cycle is not entirely detrimental. After entering the G1 phase and before progressing to the G2/M phase, podocytes undergo hypertrophy without division. This hypertrophy can partially compensate for the loss of podocytes detaching from the basement membrane.^[^ ^31^ ^]^ However, upon entering the G2/M phase or even before that, podocytes enter a fragile state while hypertrophying, making them susceptible to apoptosis or detachment from the basement membrane.^[^ ^31^ ^]^ Therefore, maintaining podocytes in a hypertrophic rather than fragile state could potentially slow down the glomerular diseases progression. A more ambitious notion is that if podocyte proliferation can be achieved in vitro, and then a sufficient number of healthy podocytes can be re‐transplanted into the body and successfully adhere to the basement membrane, it could offer more therapeutic options for patients with chronic kidney diseases.

In summary, our study revealed that upregulation of lncRNA evf‐2 expression in DN podocytes can modulate cell cycle and inflammation through its interaction with hnRNPU, affecting various processes including transcriptional activation, alternative splicing, RNA stability, and protein translation. Elucidating the molecular mechanisms underlying podocyte re‐entry into the cell cycle will aid in identifying new pathways to delay podocyte injury and discover novel therapeutic targets.

## 4 Experimental Section

### Human Renal Biopsy Samples and Clinical Data

Human renal biopsy samples, clinical data, and urine specimens were sourced from the BIO‐Bank at the First Affiliated Hospital of Zhengzhou University and the National Human Genetic Resources Sharing Service Platform (2005DKA21300). A total of 9 renal tissue samples (3 DN, 3 DM, and 3 normal controls (CON)) were randomly selected from archived biopsy tissues. Normal control kidney tissues were acquired from preimplant biopsy samples or kidneys ineligible for transplantation due to vascular anomalies. The study received approval with an exempt status for human subjects from the Institutional Ethics Committee of The First Affiliated Hospital of Zhengzhou University, and the requirement for informed consent from participants was waived (Ethics Approval No. 2021‐KY‐004‐02).

### Generation of Podocyte‐Specific evf‐2 Knockout Mice

The floxed evf‐2 mice were generated utilizing a loxP system targeting exon 3 of evf‐2. Following the acquisition of a homozygous mouse with systemic conditional evf‐2 knockout, it was bred with Nphs2‐cre mice, which possess a C57Bl/6j background and contain Cre recombinase and the Nphs2 promoter fragment. This breeding strategy yielded evf‐2 ^flox/flox, Nphs2‐cre^ mice (evf‐2 KO). Alternatively, evf‐2 ^flox/+^ mice were directly crossed with Nphs2‐cre mice to generate evf‐2 ^flox/+, Nphs2‐cre^ mice, and subsequent self‐breeding resulted in evf‐2 ^flox/flox, Nphs2‐cre^ mice, eventually leading to evf‐2 KO mice. Homozygous evf‐2 KO mice were selected for subsequent experiments, and their kidney phenotype closely resembled that of wild‐type mice. The detailed knockout methods were presented in Supporting Information.

### STZ/HFD‐Induced DN in Mice

Six‐week‐old male mice were fed a high‐fat diet (HFD) for a duration of 4 weeks (MD12032, Medicience, China) and subsequently randomly assigned to groups for further experiments. At nine weeks of age, the mice received intraperitoneal injections of streptozotocin (STZ), (55 mg per kg body weight daily for 5 consecutive days, S8050, Solarbio, Beijing, China), after which the high‐fat diet was replaced with a regular diet. Prior to the STZ injection, the animals underwent a 12 h fasting period with access to water.

### LPS Treated Mice

Ten‐week‐old control mice and evf‐2 KO mice were selected. The body weight before injection was recorded, after which each mouse received an intraperitoneal injection of lipopolysaccharides (LPS), (L8880, Solarbio) at a dosage of 10 µg per gram of body weight. The status of the mice in both groups was observed, and their body weight was recorded at 24 h after the injection. The mice were then sacrificed.

### Podocyte‐Specific evf‐2 Knockdown or Overexpression Mice

Lentiviruses containing a mouse evf‐2 fragment (exon 3) or specific shRNA targeting evf‐2 for overexpression or knockdown were synthesized by Hanbio (Shanghai, China). These lentiviruses were administered to mice via tail vein injections (single injection, virus titer 10^8^ TU/ml; 100 µl per mouse). Detailed sequences of the lentiviruses can be found in the Supporting Information. Both the overexpression and shRNA lentiviruses were driven by the podocin (Nphs2) promoter, serving as a marker for podocytes. The control lentivirus, harboring an HA tag, also contained the podocin promoter. The transfection efficiency of the podocyte‐specific promoter in glomerular podocytes was evaluated through immunofluorescence staining, revealing HA tag and WT‐1 expression in OCT‐embedded frozen kidney tissues (Figure S1, Supporting Information).

For the experimental timeline, six‐week‐old mice were fed a high‐fat diet for 4 weeks, followed by intraperitoneal injections of STZ (55 mg kg^−1^ body weight once daily for five days) at nine weeks of age. Subsequently, the mice received virus injections at ten weeks of age and were sacrificed at 22 weeks old.

All mice studies were performed according to the National Institutes of Health (NIH) guidelines for animal experiments^[^ ^45^ ^]^ and under the review and approval of the Ethics Committee of The First Affiliated Hospital of Zhengzhou University (Ethics Approval No. KY‐2021‐00712).

### Isolation of Glomeruli and Primary Culture of Podocytes

Glomeruli were isolated following established protocols,^[^ ^16^ ^]^ revealing an ≈80% decapsulation rate. Kidney tissues were stored at 4 °C throughout the isolation process, except during collagenase digestion at 37 °C. The isolated and enriched glomeruli were then cultured on collagen type I‐coated dishes at 37 °C in RPMI 1640 medium (Thermo Fisher Scientific) supplemented with 10% fetal bovine serum (Thermo Fisher Scientific), 0.075% sodium pyruvate (Sigma–Aldrich), 100 U ml^−1^ penicillin, and 100 µg ml^−1^ streptomycin (Penicillin‐Streptomycin Solution, PB180120, Procell, Wuhan, China), then was maintained in a humidified incubator with 5% CO_2_.

### Morphological Studies

Following previously established procedures,^[^ ^16^ ^]^ formalin‐fixed and paraffin‐embedded kidney tissues underwent histopathological analysis and Periodic Acid‐Schiff (PAS) staining. Kidney cortex samples fixed with 2.5% glutaraldehyde were utilized for electron microscopy (EM). Ultrathin sections were dissected and mounted on copper grids using an EM UC7 Ultramicrotome (Leica, West Hollywood, CA, USA). Micrographs were captured with an EM‐10 microscope (Carl Zeiss, Oberkochen, Germany) at 80 kV. A single observer, blinded to the conditions, meticulously examined the morphology of all sections. The pathological features or the position of podocytes were determined by pathologists.

### Immunofluorescent Staining

Immunofluorescence analyses were performed on OCT‐embedded or formalin‐fixed and paraffin‐embedded kidney tissue specimens, cultured podocytes, and primary isolated podocytes. Fixation in cold methanol lasted for 40 min, followed by blocking with 5% bovine serum albumin (BSA). Subsequently, specimens were incubated overnight at 4 °C with antibodies against B7‐1 (Santa Cruz Biotech), Podocin (Abcam), Mcp‐1 (Abcam), Claudin‐1 (Abcam), and synaptopodin (SYNPO, Proteintech). Secondary antibodies conjugated with Alexa Fluor 488 or 594 (Invitrogen, Carlsbad, CA, USA) were then applied and incubated at 37 °C for 1 h. Sections were counterstained with 4,6‐diamidino‐2‐phenylindole (DAPI, Vector Laboratories, Burlingame, CA, USA) and visualized using a Zeiss LSM 880 confocal microscope (Carl Zeiss, Oberkochen, Germany).

### Immunohistochemistry Staining

Formalin‐fixed, paraffin‐embedded kidney tissues were sectioned into 3‐µm slices. Immunohistochemistry staining was conducted using the universal SP test Kit (SP0041, Solarbio) with primary antibody against PCNA (Abcam). Images were captured using the Olympus BX53F microscope (Tokyo, Japan).

### RNA Fluorescence In Situ Hybridization (FISH) with Immunofluorescence

*FISH experiment of human and mouse kidney*: Renal RNA fluorescence in situ hybridization (FISH) was carried out in combination with immunofluorescence. The Custom LNA LncRNA FISH Detection Probes & Kits (QIAGEN, German) was used with a double digoxin‐labeled nucleic acid strand probe (human lncRNA EVF‐2: 5DigN/TTGCCTGTTCCATATCAATT/3DigN; mouse lncRNA evf‐2: 5DigN/TGTTAAGTGAGACAGGCATTCA/3Dig_N), according to the manufacturer's instructions.

*FISH experiment of isolated primary podocytes*: FISH detection of lncRNA evf‐2 in isolated primary podocytes were accomplished using a FISH detection kit and probe for lncRNA evf‐2 (C10190‐FISH Kit and lnc1100312, Ribobio, Guangzhou, China) according to the manufacturer's instructions.

### Cell Culture and Treatments

MPC5 cells were acquired from ATCC /Shanghai Cell Bank (BLUEFBIO, number BFN60808809). Conditionally immortalized MPC5 cells (passages5‐10) were cultured as reported previously.^[^ ^16^ ^]^ To determine the effects of overexpressing lncRNA evf‐2 on podocytes cultured under NG concentration (NG, 5.6 mmol L^−1^ D‐glucose), cultured normal mouse podocytes were randomly divided into three groups: NG, control adenovirus (NG + con‐AD) and evf‐2‐overexpressed adenovirus (NG + evf‐2).

To determine the effects of hnRNPU‐siRNA on podocytes overexpressing evf‐2, cultured mouse podocytes were randomly allocated into two groups: siRNA NC (evf‐2 + NC), and hnRNPU‐siRNA (evf‐2 + U‐siRNA).

The lncRNA evf‐2 (full length)‐overexpressing adenovirus (detailed sequences see the *Supporting information)* used in this study were designed and synthesized by Hanbio (Shanghai, China). Podocytes were seeded in 6‐well plates at 60%–70% confluence and infected with adenovirus following the manufacturer's instructions (4×10^6^ podocytes; MOI = 100). The media was replaced with fresh ones after 6–8 hrs of viral infection. The control adenovirus (NG + con‐AD), carrying an EGFP tag, transfection efficiency of adenovirus in MPC was shown in Figure S2 (Supporting Information).

The siRNA (detailed sequences see the Supporting information) used in this study were designed and synthesized by Hanbio (Shanghai, China). Cells were cultured in serum‐free medium for 12 hrs before siRNA transfection, and transfected with Lipofectamine RNAiMAX transfection reagent (13 778 075, Invitrogen) for 4–6 h before changing the medium. The siRNA concentration was 75 nM.

### SDS‐PAGE and Western Immunoblot Analysis

Lysing and homogenization of isolated renal glomeruli or cultured podocytes were carried out using radio‐immunoprecipitation (RIPA) buffer (R0010, Solarbio) containing protease inhibitors (CW2200S, CWBIO) and phosphatase inhibitors (CW2383S, CWBIO), PMSF (P0100, Solarbio). The protein content was quantified, and immunoblotting was performed following established protocols. Protein markers (26 616, Thermo Fisher Scientific) were used for verification. Antibodies against B7‐1 (Abcam), Podocin (Abcam), Mcp‐1 (zenbio), Claudin‐1 (Abcam), hnRNPU (Proteintech) respectively, while those against GAPDH were obtained from Goodhere Biotechnology (Hangzhou, China). Secondary antibodies were acquired from Abbkine Biotechnology (A21020, Wuhan, China). The relative grayscale of band blots was quantified using ImageJ software 10.0.

### RNA Extraction and Real‐Time Reverse Transcription‐PCR

Total RNA was extracted from cultured cells and tissues using the RNeasy Mini Kit (74 104, QIAGEN, Germany) following the manufacturer's protocol. The Eppendorf Biospectrometer spectrophotometer was employed to assess RNA concentration and purity. Subsequently, RNA was diluted to the concentration of 300 ng/µl in RNA‐free water. Complementary DNA (cDNA) was synthesized through reverse transcription using the RevertAid First Strand cDNA Synthesis Kit (K1622, Thermo Fisher Scientific). Oligo (dT) primer was utilized for messenger RNA, while random primer was employed for lncRNAs, following the manufacturer's instructions. Quantitative Real‐time PCR (qRT‐PCR) was conducted to measure the relative expression levels of target genes using the THUNDERBIRDNext SYBR qPCR Mix (QPX‐201, Toyobo, Japan). Primers for mRNA and lncRNAs were designed and synthesized by Thermo Fisher Scientific and were listed in the Supporting Information. All experiments were performed in triplicate, with the amplification signals of GAPDH mRNA serving as the internal control.

### Flow Cytometric Cell Cycle Analysis

Flow cytometry was performed according to the manufacturer's protocol of cell cycle and apoptosis detection kit (C1052, Beyotime, Shanghai, China). Podocytes were harvested and fixed with 70% ethanol for 12 h at 4 °C. Following fixation, cells were centrifuged at 1000 × g, washed with PBS, and then stained with propidium iodide for 30 min at 37 °C in the dark. Cell cycle analysis was conducted using a FACSCelesta flow cytometer (Becton Dickinson) at an excitation wavelength of 488 nm, and the data were analyzed using FlowJo software (FlowJo, Ashland, USA).

### RNA Sequencing

RNA‐seq analysis was conducted on podocytes overexpressing lncRNA evf‐2 under normal conditions, and the obtained results were compared with those from normal controls. The RNA‐seq experiments and data analysis followed previously described methods.^[^ ^16^ ^]^ Differentially expressed genes were identified through statistical analyses (fold change > 1.5, and *p* < 0.05). Subsequent data‐mining analyses included correlation analysis, clustering, and Gene Ontology (GO) analysis and Kyoto Encyclopedia of Genes and Genomes (KEGG) pathway analyses.

### Chromatin Isolation by RNA Purification (ChIRP) coupled with Mass Spectrometry (MS)

The ChIRP‐MS analysis was performed by Aksomics Inc. (Shanghai, China). The cells were cross‐linked with 3% formaldehyde for 30 min, the cells were lysed by sonication, and then the target lncRNA evf‐2 was enriched by the biotin‐labeled antisense oligonucleotide. The interacting proteins were eluted and separated by electrophoresis, and the bands at each position could be identified by LC/MS‐MS.

### Chromatin Immunoprecipitation (ChIP)

The immunoprecipitated DNA was analyzed through deep sequencing (ChIP‐seq) by SEQHEALTH Biotech Company (Wuhan, China). 1 × 10^7^ cells underwent fixation and cross‐linking with formaldehyde at room temperature for 10 min. Subsequently, lysis buffers were employed to break down the cells, and sonication was conducted to shear the cross‐linked chromatin and proteins into appropriately sized fragments (200‐1000 bp). The sheared DNA underwent 30 cycles of sonication (10 sec on and 10 sec off) using the Biorupter Pico (Diagenode, Belgium). After centrifugation at 10 000 × g at 4 °C for 10 min, the supernatant containing fragmented DNA was collected and prepared for immunoprecipitation. Three tubes of sheared DNA were combined with 20 µl of fully resuspended protein A/G magnetic beads, along with the positive control (Anti‐RNA Polymerase II), the negative control (normal Rabbit IgG), and the antibody against hnRNPU (Proteintech). Following overnight incubation at 4 °C with rotation, the protein/DNA complexes were eluted, and the free DNA was extracted for ChIP‐seq analysis.

### RNA Binding Protein Immunoprecipitation (RIP)

The enrichment of hnRNPU was assessed using a Magna RIP RNA‐Binding Protein Immunoprecipitation Kit (17‐700, Millipore, Germany) following the manufacturer's protocol. Briefly, 1 × 10^7^ cells were lysed in RIP lysis buffer, followed by immunoprecipitation with antibodies (against Rabbit IgG or against hnRNPU) using protein A/G magnetic beads. The supernatant of the lysis buffer was added to each beads‐antibody complex, and the mixture was incubated overnight at 4 °C with rotation, immobilized magnetic beads bound complexes with magnet and washed off unbound materials. After the incubation, RNA extraction was performed, and the RNA was subsequently analyzed by qRT‐PCR or sequencing.

### AlphaFold Prediction

AlphaFold was a machine learning‐based protein structure prediction program, which had been used to accurately predict the structures of proteins. Herein, the structure of hnRNPU protein was predicted by AlphaFold.^[^ ^46^, ^47^ ^]^ Sequences for the human reference proteome were obtained from UniProt release 2023_03.^[^ ^48^ ^]^ This predicted hnRNPU protein consists of four functional regions.^[^ ^49^ ^]^

### Statistical Analyses

Data and statistical analyses were performed using SPSS V22.0 (IBM Corporation, Armonk, New York, USA). The data were expressed as the mean ± SD. The unpaired t‐test was used to compare two groups, and ordinary one‐way or two‐way ANOVA was used to compare multiple groups, followed by the Dunnett's multiple comparisons test, Tukey's multiple comparisons test, or Sidak's multiple comparisons test, using GraphPad Prism 8.0 software (GraphPad Software, San Diego, CA, USA). Linear regression analysis was used to examine possible relationships between the two parameters. A value of *p* < 0.05 was considered statistically significant.

## Conflict of Interest

The authors declare no conflict of interest.

## Author Contributions

C.Z. performed the experiments and wrote the manuscript. H.Z. analyzed patient samples and revised the manuscript. Y.Y. performed some in vivo experiments and advised on the manuscript. Y.L. prepared reagents and protocols and carried out some in vivo experiments. M.L. and Y. L. performed some of the cell‐based experiments and helped with animal maintenance. L.Y. and H. Z. performed the prediction of hnRNPU protein structure by using AlphaFold. S.Z. and S.P. helped select and prepare the biopsy samples of patients and interpret the images. Z.L. supervised the experimental design and data interpretation. J.G. designed and conducted the experiments, analyzed and interpreted data, and wrote the manuscript. All authors contributed to and approved the manuscript.

## Supporting information



Supporting Information

## Data Availability

The data that support the findings of this study are available from the corresponding author upon reasonable request.
