# Supplementary material for: LncRNA evf‐2 Exacerbates Podocyte Injury in Diabetic Nephropathy by Inducing Cell Cycle Re‐entry and Inflammation Through Distinct Mechanisms Triggered by hnRNPU
Source: Adv Sci (Weinh). 2024 Oct 29;11(47):2406532. doi: 10.1002/advs.202406532 (PMC11653703; doi:10.1002/advs.202406532)

ADVANCED SCIENCE
Open Access

Supporting Information

for *Adv. Sci.*, DOI 10.1002/advs.202406532

LncRNA evf-2 Exacerbates Podocyte Injury in Diabetic Nephropathy by Inducing Cell Cycle Re-entry and Inflammation Through Distinct Mechanisms Triggered by hnRNPU

Chaojie Zhang, Hui Zhao, Yufan Yan, Yanfei Li, Min Lei, Yong Liu, Longhua Yang, Huijian Zhao, Sijie Zhou, Shaokang Pan, Zhangsuo Liu and Jia Guo**

Supporting information:

1. Supplementary Figures and Tables
2. Supplementary Methods
3. The orginal uncropped images of western blot

1. Supplementary Figures and Tables

Supplementary Figure. S1

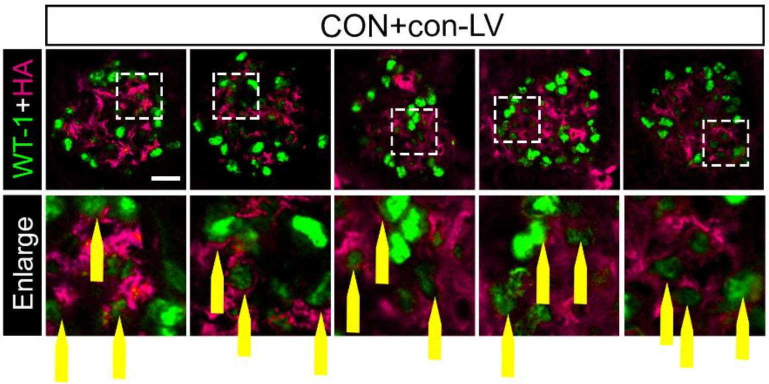

Supplementary Fig. S1. Immunofluorescence staining showing the expression of HA-tag and podocyte marker protein WT-1 (expressed in the nuclear) in the OCT-embedded frozen kidney tissue of mice. Bar=20μm.

Supplementary Figure. S2

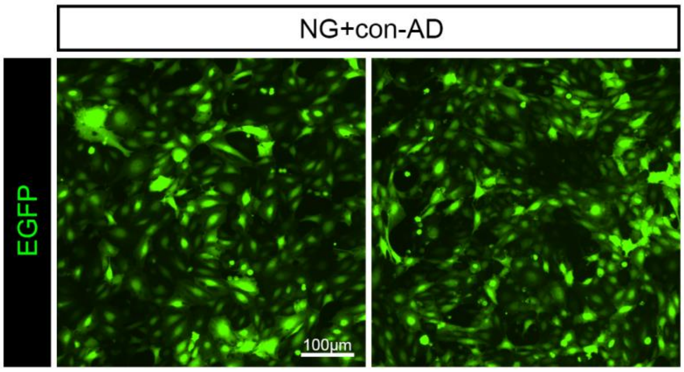

Supplementary Fig. S2. The transfection efficiency of adenovirus in MPC.

Supplementary Figure. S3

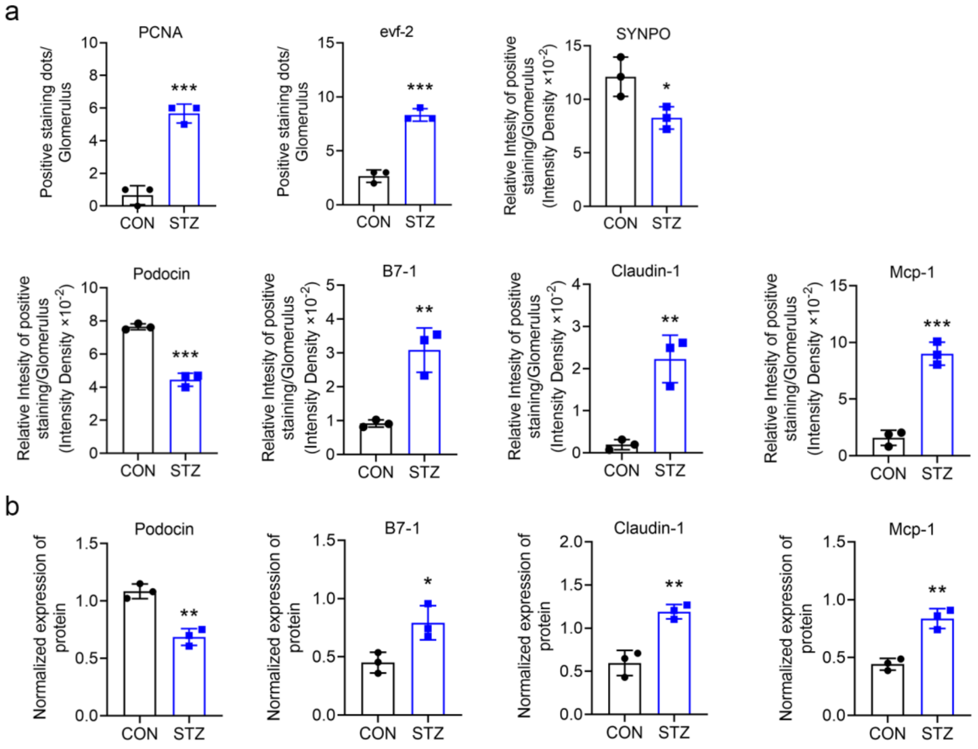

Supplementary Fig. S3. The intensity of the immunofluorescence staining and western blot shown in Fig.1 is quantified via ImageJ software and compared in the different samples.

a. The intensity analysis of Fig.1f-g. n = 3.

b. The intensity analysis of Fig.1h. n = 3. *P < 0.05, ** < 0.01, *** < 0.001, (unpaired t-test).

Supplementary Figure. S4

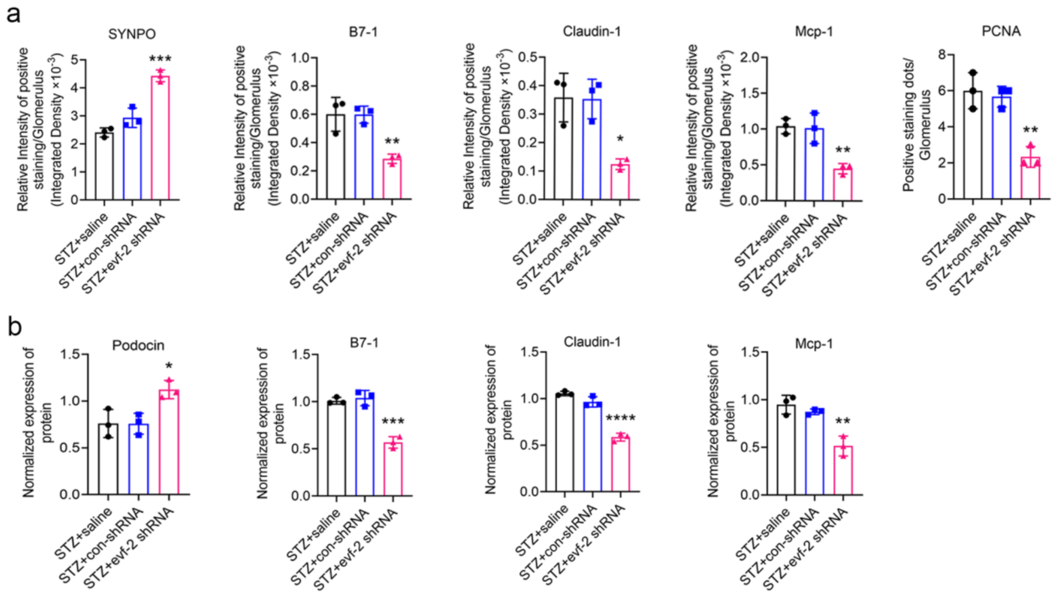

Supplementary Fig. S4. The intensity of the immunofluorescence staining and western blot shown in Fig.2 is quantified via ImageJ software and compared in the different samples.

a. The intensity analysis of Fig.2h. n = 3.

b. The intensity analysis of Fig.2i. n = 3. **P* < 0.05, ** < 0.01, *** < 0.001, **** < 0.0001, (one-way ANOVA plus Tukey's multiple comparisons test).

Supplementary Figure. S5

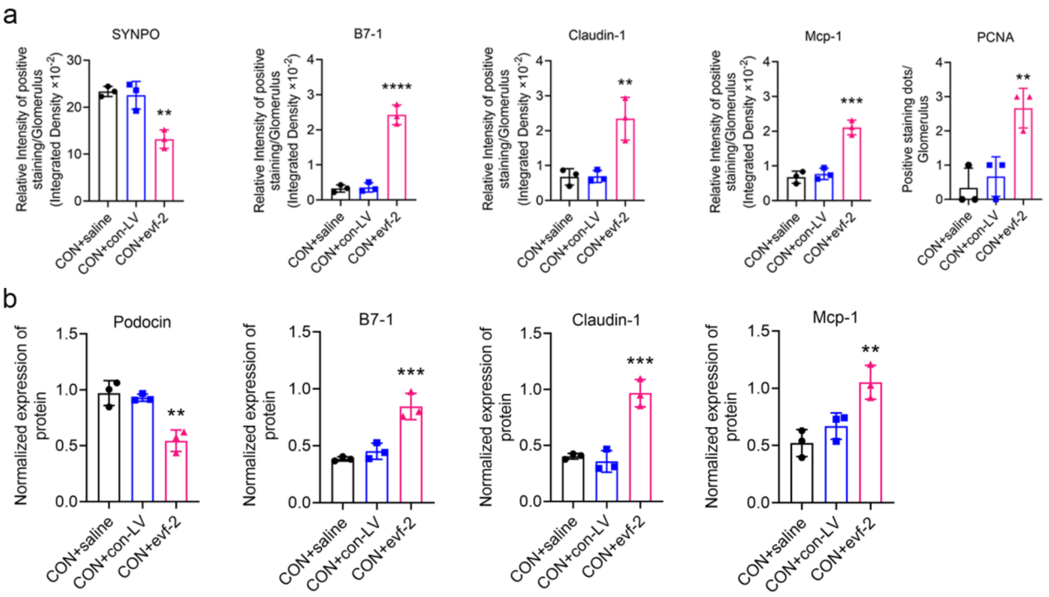

Supplementary Fig. S5. The intensity of the immunofluorescence staining and western blot shown in Fig.3 is quantified via ImageJ software and compared in the different samples.

a. The intensity analysis of Fig.3h. n = 3.

b. The intensity analysis of Fig.3i. n = 3. ***P* < 0.01, *** < 0.001, **** < 0.0001, (one-way ANOVA plus Tukey's multiple comparisons test).

Supplementary Figure. S6

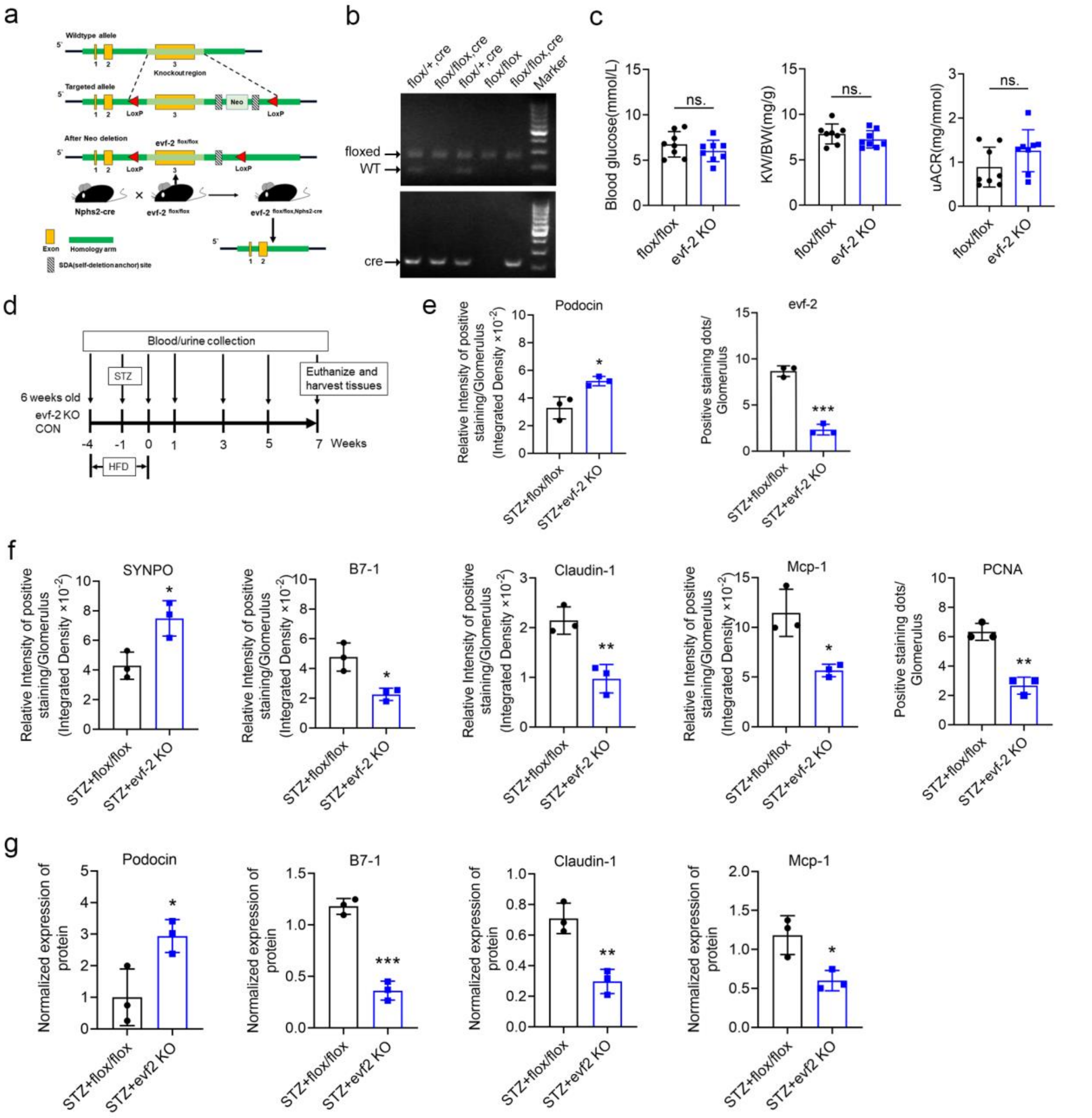

Supplementary Fig. S6. The supplementary figures for STZ-treated evf-2 KO mice.

a. A flow chart illustrating the preparation of evf-2-specific knockout mice.

b. Verification of gene knockout in knockout mice (evf-2 KO). Positive bands for both floxed and cre indicate successful knockout, while the absence of the wild-type gene band confirms podocyte-specific knockout of evf-2.

c. Comparative analysis of random blood glucose levels (RBG), kidney weight/body weight (KW/BW) ratio, and urinary albumin/creatinine ratio (uACR) between the two groups of mice. n = 8 mice/group.

d. A flow chart depicting the streptozotocin (STZ)-mediated induction of diabetes in evf-2 KO mice.

e. The intensity analysis of Fig.4h. n = 3.

f. The intensity analysis of Fig.4i. n = 3.

g. The intensity analysis of Fig.4k. n = 3. *$P < 0.05$, ** < 0.01, *** < 0.001, (unpaired t-test).

Supplementary Figure. S7

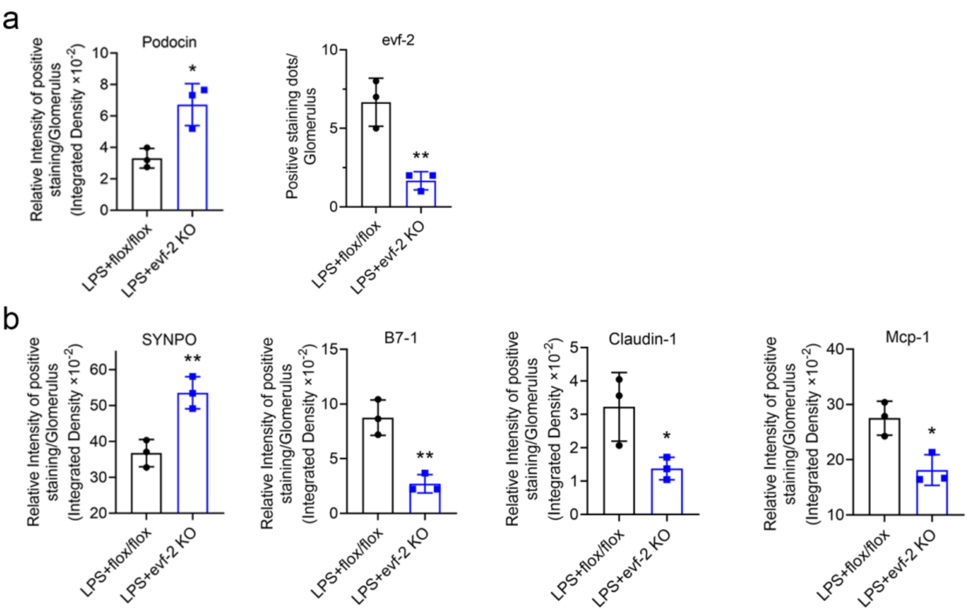

Supplementary Fig. S7. The intensity of the immunofluorescence staining shown in Fig. 5 is quantified via ImageJ software and compared in the different samples.

a. The intensity analysis of Fig.5g. n = 3.

b. The intensity analysis of Fig.5h. n = 3. *P < 0.05, ** < 0.01, (unpaired t-test).

Supplementary Figure. S8

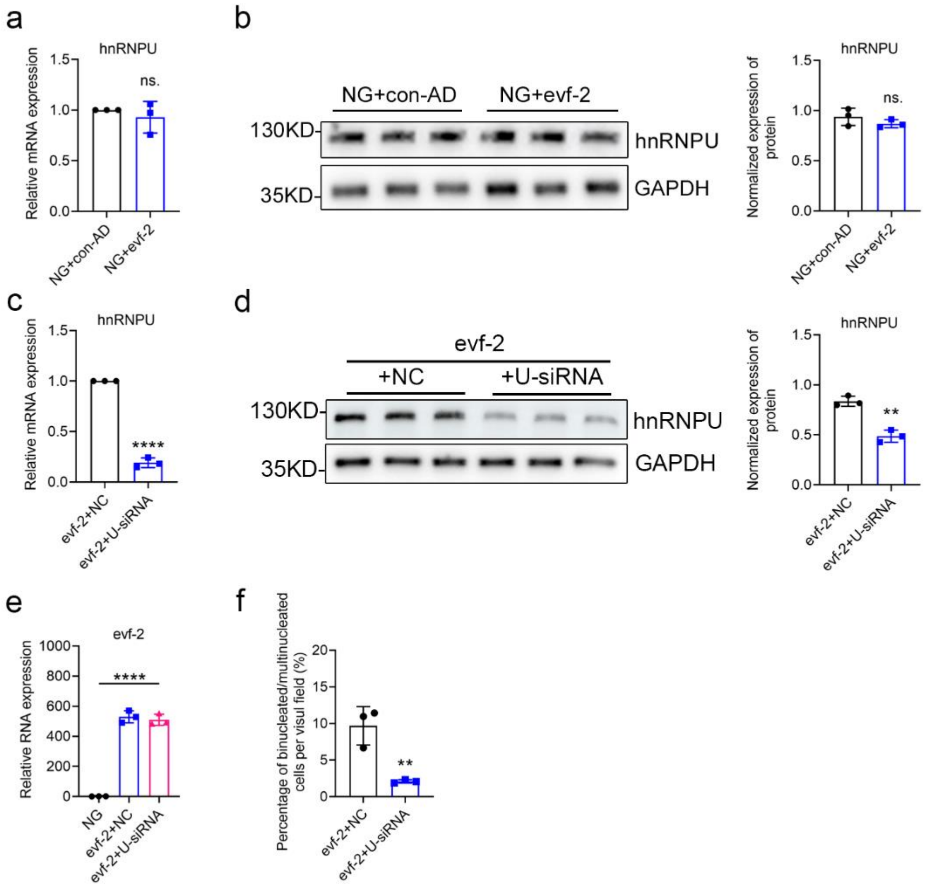

Supplementary Fig. S8. The supplementary figures for MPC transfecting hnRNPU siRNA.

a, b. Quantitative real-time polymerase chain reaction (qRT-PCR) and western blotting demonstrated the impact of evf-2 overexpression on hnRNPU' mRNA and protein expressions.

c, d. qRT-PCR and western blotting revealed the transfection effects of hnRNPU siRNA.

e. The expression of evf-2 was assessed by qRT-PCR in podocytes under normal conditions (NG, 5.6 mM), overexpression of evf-2 plus NC siRNA transfection (evf-2 + NC), and overexpression of evf-2 plus hnRNPU siRNA transfection (evf-2 + U-siRNA). ****$P < 0.0001$, evf-2 + U-siRNA/evf-2 + NC vs NG, (one-way ANOVA plus Tukey's multiple comparisons test).

f. The quantitative analysis of Fig.8c. **$P < 0.01$, **** < 0.0001, (unpaired t-test).

Supplementary Figure. S9

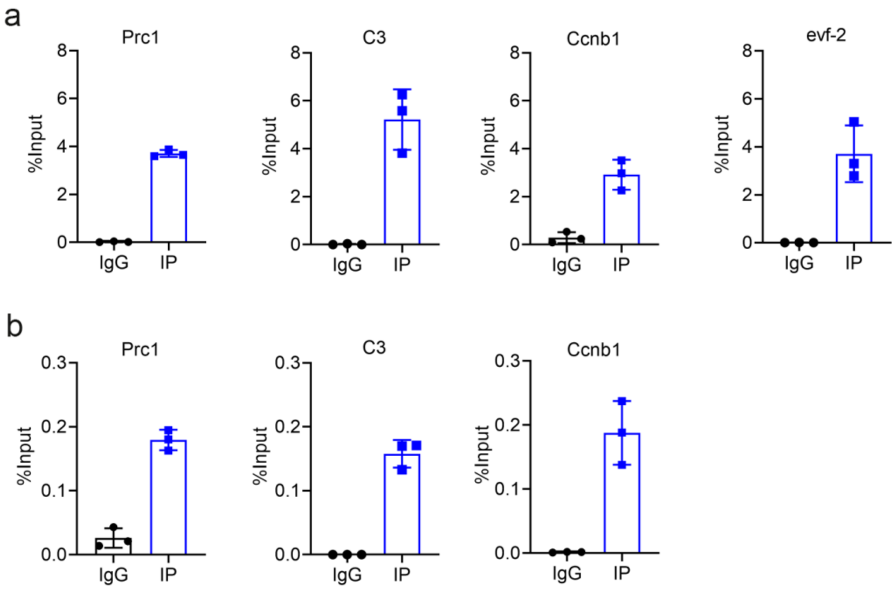

Supplementary Fig. S9. qRT-PCR was employed to confirm the results of the binding genes identified in RIP (a) and ChIP (b) assays.

Table **S**1: Primers sequences for qRT-PCR

Prc1	F:GAGCAGAGGCTACAAAGAACCG<br>R:GGATGTCACGGAGTTCTTGCT
Ccnd1	F:AGGCGGATGAGAACAAGCAG<br>R:AAGAAAGTGCGTTGTGCGGTA
Ccnb1	F:TGCCTCTGCACTTCCTCCGT<br>R:ATGCACCATGTCGTAGTCCAGC
Nek2	F:CTTGGGCTGCCTGCTGTATG<br>R:GAGGTCATTCAAGCCATCAGAGT
Ccnf	F:GAAGTGTTTCCACGATGATGC<br>R:GCTCTGGGCTTTCCTGTTTT
Cd80	F:CTTTCAGACCGGGGCACATA<br>R:ATGGGTTTCCAGACTCAGTTATGTT
Ccl20	F:GTGGGTTTCACAAGACAGATGG<br>R:ACAGCCCTTTTCACCCAGTTC
C3	F:TTGAGGTGAAGGAATACGTGCTG<br>R:AGGAACTTGGCTATGATGGAAACT
Lcn2	F:ACAGAAGGCAGCTTTACGATGT<br>R:ACTGGTTGTAGTCCGTGGTGG
Ccl2	F:AGGTCCCTGTCATGCTTCTGG<br>R:AACTACAGCTTCTTTGGGACACCT
hnRNPU	F:TCCTCAGCCACCTGTTGAAG<br>R:GCCTTTTGACACACCGTAGG
evf-2	F:AGAAACCTACGCCCAGAAGAAC<br>R:GTGGCTCATAAGACTGGACAGC

Table S2: The catalog numbers of antibodies

Antibody	Catalog Number
Podocin	Abcam, ab50339
Synaptopodin (SYNPO)	Proteintech, 21064-1-AP
B7-1	Santa Cruz Biotech, sc376012
Mcp-1	Zenbio, 507277
Claudin-1	Abcam, ab180158
GAPDH	Goodhere Biotechnology, AB-P-R001
hnRNPU	Proteintech, 14599-1-AP
PCNA	Proteintech, 10205-2-AP
HRP, Goat Anti-Rabbit IgG	Abbkine Biotechnology, A21020

2. Supplementary Methods

- ◆ Sequences of lentivirus (overexpression and knockdown)
- ◆ Sequences of adenovirus
- ◆ Sequences of siRNA
- ◆ Calculation process of qRT-PCR results for verifying ChIP and RIP
- ◆ Podocyte-specific knockout of lncRNA evf-2 in mice.

◆ Sequences of lentivirus (overexpression and knockdown)

evf-2(overexpression):

exon3:

GCTCCAGTGGCCAGTTTCAAAATACCCTCCCTTTTGATGTTAGGTTACATAAACATTGTT
CTTTTTTAGGGAGGGTCTCTTTTATCAACTTTTAAAAACACACATCAGGTTCTCTGGTAT
TAAAAAGATGCCATCTCTGAGTCCCCTACTATCTGTGCTGCCTGCCTTTCCTCCTGTTCT
TTCCTTATTCCCATCCCTATTGAACTTGTGCTATGCAGTATGCATCAGGTATGTGTTAGCT
TTGGGGATACATGATAGATAAACTGGACACACAGGGTCTTCCCATTCTCTTCTGGAATT
TTCTTTGGAGGGAGCCTCTTGTATCTAGACAGACCGTGCTGTGGTACCCCAGAGGTAA
CCACCTACAGGCTTCACTCTGCCTAAGCAATTTTGCTGTGCACTAAGATACACATTCAA
GTAACTTTAGATTACCACAATAACTTTCTCCAGGTATGAGGAAAAGAGATAATTTACTTC
TGAGATGTGTATAGGATAGCCCTCCATCCTGGGAAGAACAGTGACTACTCCCTGCATCC
CGACCTTGCCCAGGGAAAGCTAATGTTTCTCTGTGTTATCCCTGTGACTTGCCACTTCT
TTAAAAAGGAATGGGCAAACAATAAACAGACAAAAATGTTGTCTGACCTCATTGGAAA
TCCTTTTAAGAATTAATCCTTTCTATCTCCTTCATTATCAACAAATCTATTGAATACTTATC
TCTGAGTCCAGGGCATATTTTATAATACATAAAACAATGGAATTTCAAAATTGGAGCACT
GACATACAATATTGGTTTTGAGTATTTTTATTATAGGGAATGACTTTAGACATTGCAATTT
ATGACTTAACTGATAAAATGGATGACTCTTGACTTTCAATTTTCATTTTCAGTTCAGTCG
AGGAATAGCTTCCTCCAGGTAATGTCTATACTTTCCTATGACTAAGGGCTCTAACTATCT
CTGTTGCTTTTCTTTATGTAGGCATATGTTAGTATTTATTTTCTATATGACAAATGTATTAA
AGAAAGCATGAAATTAATGAGATAAACTTTTCAGATAGGAGTTTAGAAAATCAAGGGG
CCAAGATAAATAAATGAAAAATCAACTTAAATAATTAACATATTCCAGATATATTGGAAT
AAATGTTTATTGTACCCTTTTGGTTTTGTCTTGGGTTATTTTTTTCTTATCTCACTGATTTT
TTTTTCTTTCCTTTTTAGCTTTTTTGTCTTTTTTGATTTTTGTTGTTGCGTTTCTCCTTTTT
TTTTTTCTTGTTGATGTTGTTTGTTTGTTTGTTTGTTTGTTTTTTGAGAAAGAACAGAAG
GTTGGTTGGATAGGGAGGTGGGGAAGATCTATCTGGATGGAGTTGGGAGGAGGGAAA
ATACACGATCAAAATATATTTTGTGATGGGCAGGGCATAGTGGTACATGTCTTTAATCTC
AGCACTCTGGAGGCAGAGGCAGGTGGATCTCTATGAGATGGAGGCTAGCCTGATATAC
AAAGTGAGACCAGAACATAGGGCTGCCTCAAAAACTTTATATATATATTAAAAATGTTT
GCTTTTTGAGACAGTCACAGATAACCAAAACTGATCTTGTAATGATGTAAACATGTCCA
GCTAATTTTCAAATATTGTAGGGCAGCATTTCTCCCTTTGTGCACACGTGGAGTCAGCA
AATCCATATAATTCTAACCATTCTGGTGAAAAGGAGAACACTCGGCCAAGCATCTCACA
CTTCCAAGTGTGAAGCCTTGTTTGAAAGCTCCGAG.

evf-2 shRNA:

The sequences of the control (con-shRNA) shRNA were as follows:

shRNA sequence
Top strand:
aattcGTTCTCCGAACGTGTCACGTAATTCAAGAGATTACGTGACACGTTCGGA
GAATTTTTTg
Bottom strand:
gatccAAAAAATTCTCCGAACGTGTCACGTAATCTCTTGAATTACGTGACACGT
TCGGAGAACg

The lncRNA evf-2 shRNA sequences were as follows:
shRNA3 sequence
Top strand:
aattcGCAGTGAATGCCTGTCTCACTTAACATTCAAGAGATGTTAAGTGAG
A CAGGCATTCACTGTTTTTTg
Bottom strand:
gatccAAAAAACAGTGAATGCCTGTCTCACTTAACATCTCTTGAATGTTAA
GT GAGACAGGCATTCACTGCg

◆ Sequences of adenovirus
evf-2(overexpression): full length.

◆ Sequences of siRNA
NC siRNA
sense strand: UUCUCCGAACGUGUCACGU TT
antisense strand：ACGUGACACGUUCGGAGAA TT

hnRNPU(mouse) -siRNA:
sense strand: CGGUGUGUCAAAAGGCAAATT
antisense strand：UUUGCCUUUUGACACACCGTT

◆ Calculation process of qRT-PCR results for verifying ChIP and RIP
Two common methods used to normalize ChIP-qPCR data include the Percent Input Method and the Fold Enrichment Method. Here we adopt the Percent Input Method.

◆ Podocyte-specific knockout of lncRNA evf-2 in mice
The mouse model was established with assistance from Cyagen Biological Company (Suzhou, China). Systemic conditional knockout of lncRNA evf-2 was established in C57BL/6J mice (evf-2 $^{flox/+}$). These mice were inbred to generate the evf-2 $^{flox/flox}$ mice, which were crossed with Nphs2-cre C57BL/6J mice (bearing the Cre recombinase and Nphs2 promoter fragment) to generate podocyte-specific lncRNA evf-2 KO mice (evf-2 $^{flox/flox,\ Nphs2\text{-}cre}$ mice). evf-2 $^{flox/+}$ mice could be

directly crossed with the Nphs2-cre mice to generate evf-2 $^{flox/+, Nphs2-Cre}$ mice, which were then inbred to obtain evf-2 $^{flox/flox, Nphs2-cre}$ mice. Podocyte-specific knockout of lncRNA evf-2 was confirmed using a routine PCR protocol to genotype tail DNA samples with the following primer pairs:

1) Neo PCR:
F1: 5 ' -TGGGAGGCTTTTGAGTTAGGAAT-3';
R1: 5 ' -ATTGGACCATCTTACTGGACAGG-3';
Target products: Wild type (WT): 222 bp; Mutant (MT): 327 bp
2) Nphs2-cre PCR:
F2: 5 ' -CGGTTATTCAACTTGCACCA-3'
R2: 5 ' -GCGCTGCTGCTCCAG-3'
Target products: Wild type (WT): NA Mutant (MT): 200 bp
3) 5'arm PCR
F3: 5 ' -TTGATGGTTTTGGTAGATTATGCCC-3'
R3: 5 ' -TGAAAAGTCAGAAGCACTGTTACC-3'
Target products: Wild type (WT): 170 bp; Mutant (MT): 257 bp

The construction of knockout mice based on the information on the ensemble website. http://asia.ensembl.org/Mus_musculus/Transcript/Summary?db=core;g=ENSMUSG00000090063;r=6:6820543-6871592;t=ENSMUST00000159568.

3. The orginal uncropped images of western blot

Primary western blotting for Fig1h.

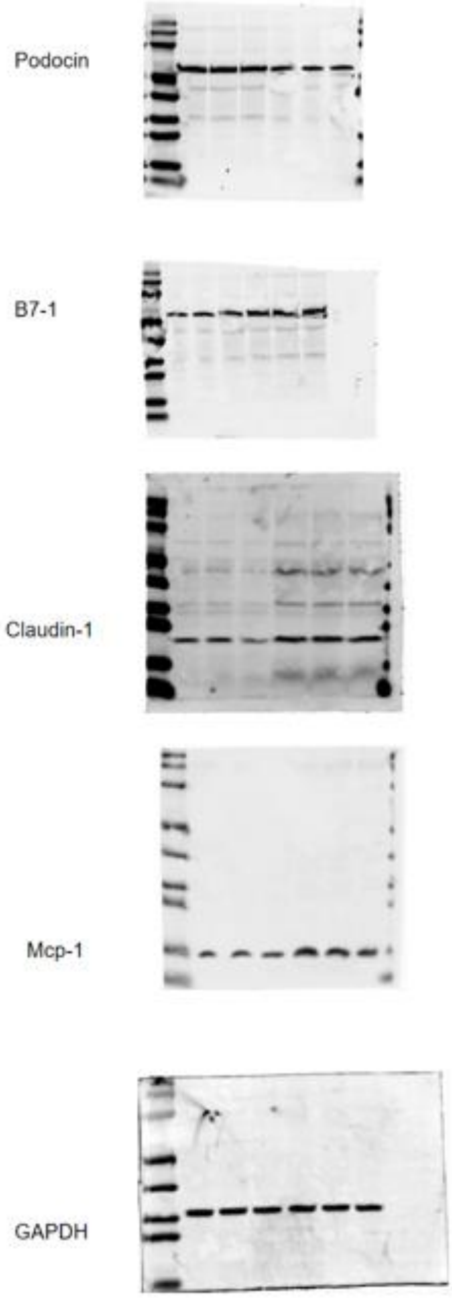

Primary western blotting for Fig2i.

Podocin

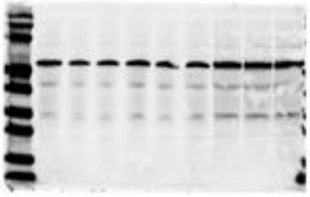

B7-1

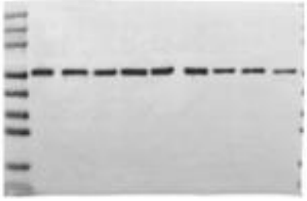

Claudin-1

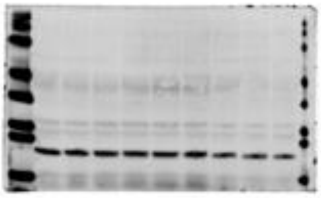

Mcp-1

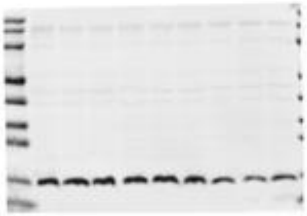

GAPDH

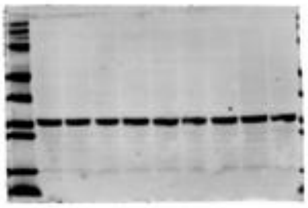

Primary western blotting for Fig3i.

Podocin

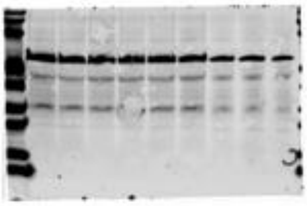

B7-1

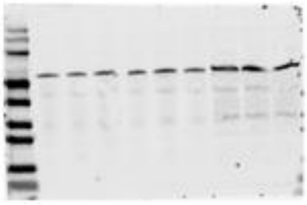

Claudin-1

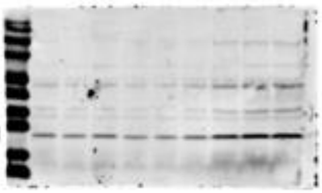

Mcp-1

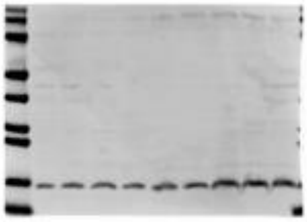

GAPDH

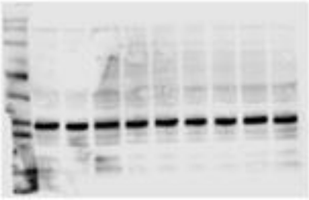

Primary western blotting for Fig4k.

Podocin

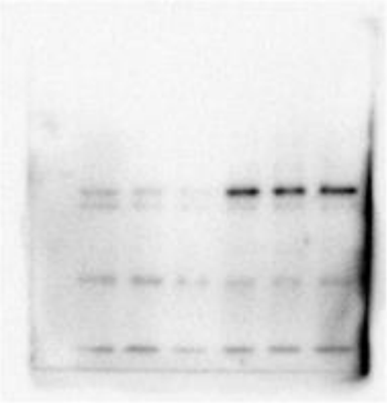

Claudin-1

GAPDH

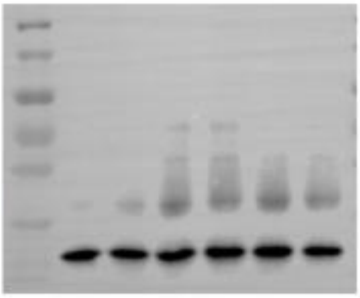

B7-1

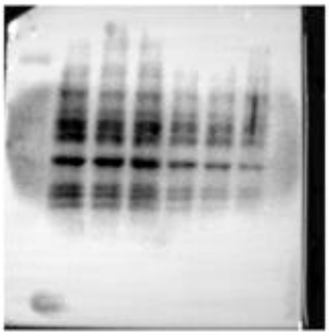

Mcp-1

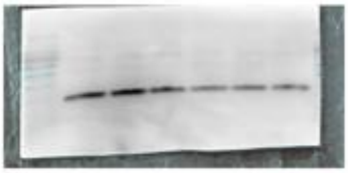

Primary western blotting for Fig5i.

Podocin

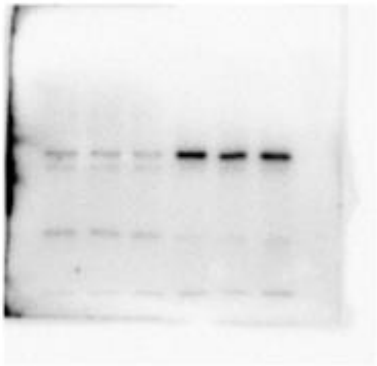

Claudin-1

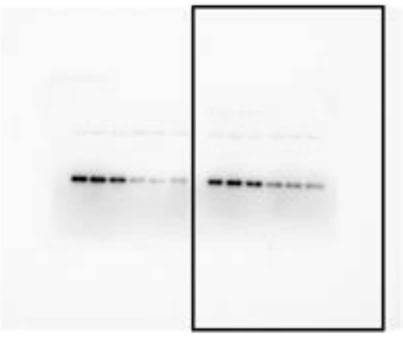

GAPDH

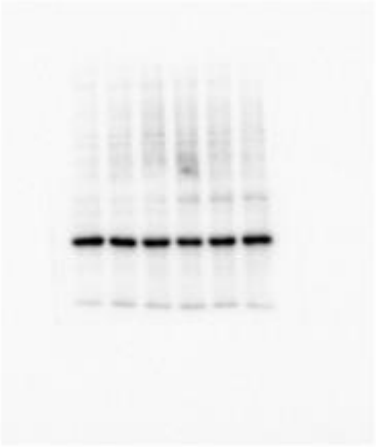

B7-1

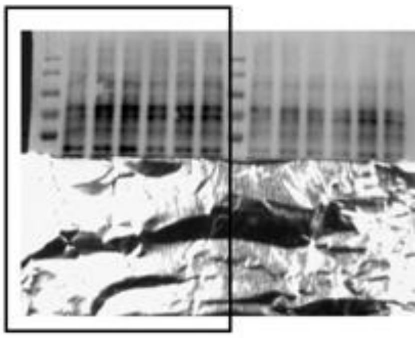

Mcp-1

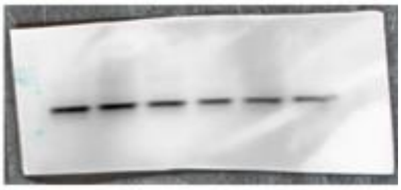

Supplement: Supplementary file 1 — Supporting Information [file ADVS-11-2406532-s001.pdf]
